# Supplementary material for: MiR-155 promotes compensatory lung growth by inhibiting JARID2 activation of CD34+ endothelial progenitor cells
Source: PLoS One. 2024 Feb 23;19(2):e0296671. doi: 10.1371/journal.pone.0296671 (PMC10890733; doi:10.1371/journal.pone.0296671)

Figure 2G

VEGF

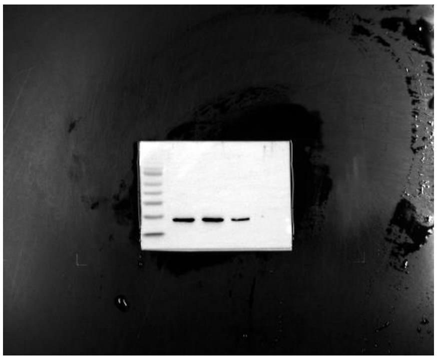

MMP-2

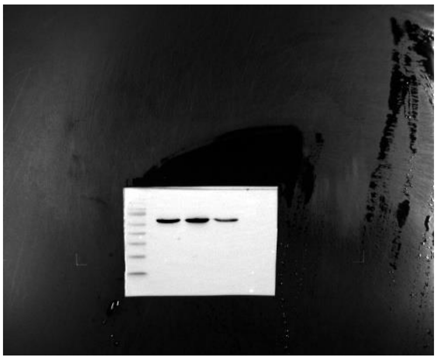

GAPDH

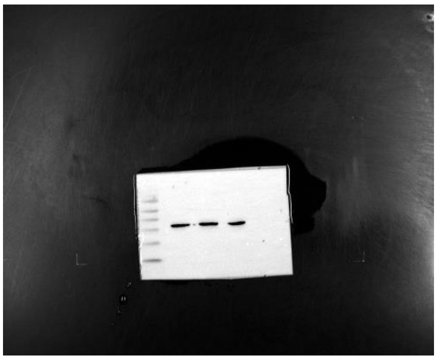

Figure 3A

JAPID2

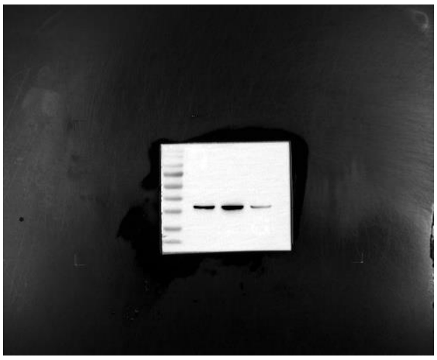

β-actin

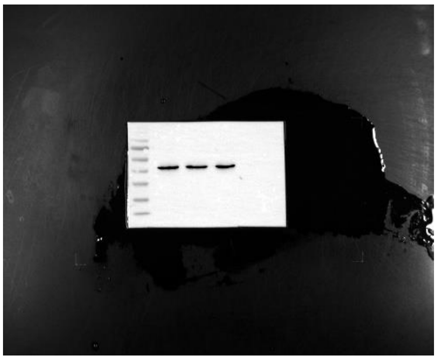

Figure 3G

VEGF

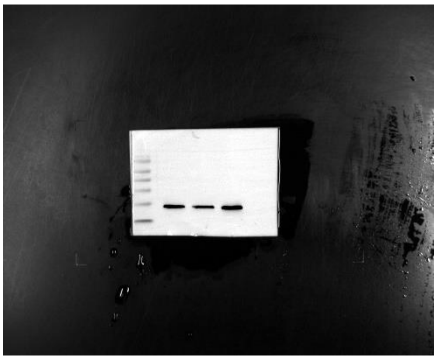

MMP-2

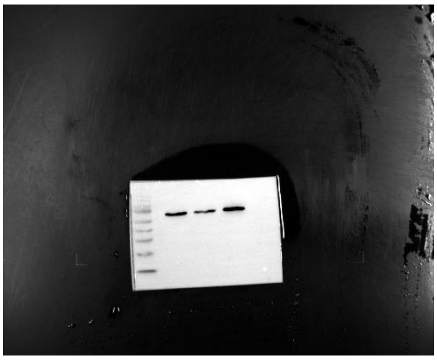

GAPDH

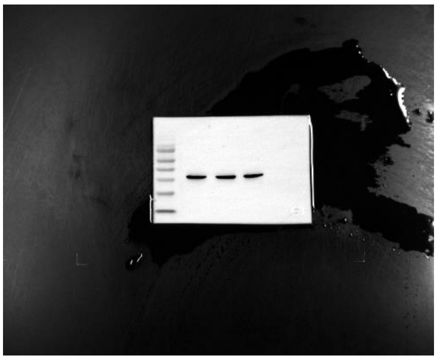

Figure 4C

JAPID2

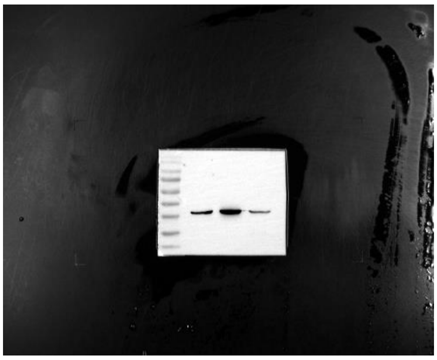

β-actin

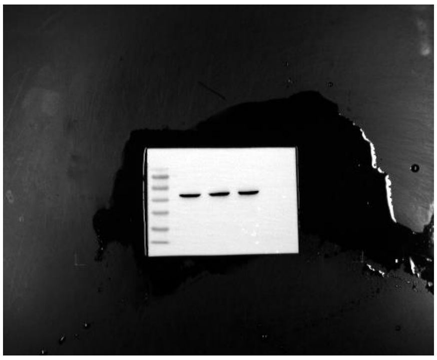

Figure 5G

VEGF

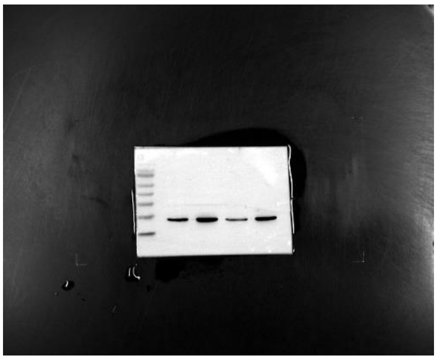

MMP-2

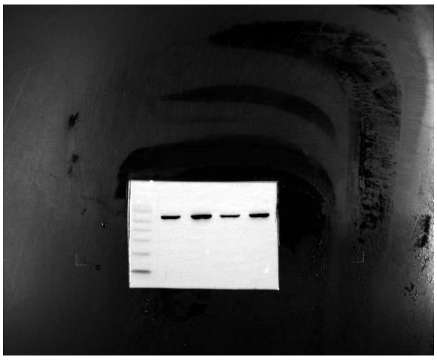

GAPDH

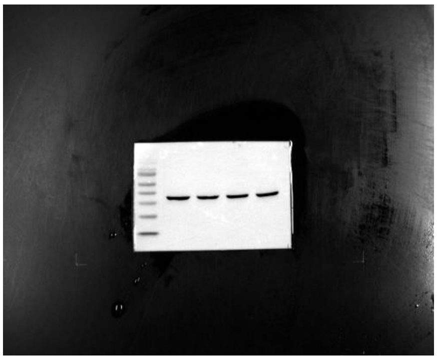

Figure 6G

VEGF

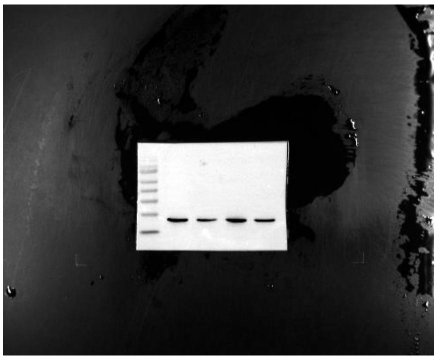

MMP-2

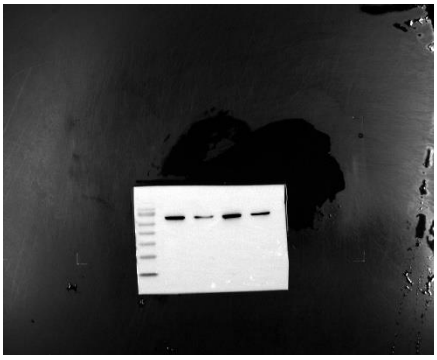

GAPDH

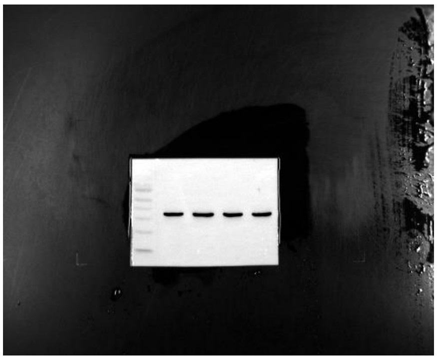

Supplement: S1 Raw images — (PDF) [file pone.0296671.s001.pdf]
