# Supplementary figures and images for: MiR-155 promotes compensatory lung growth by inhibiting JARID2 activation of CD34+ endothelial progenitor cells
Source: PLoS One. 2024 Feb 23;19(2):e0296671. doi: 10.1371/journal.pone.0296671 (PMC10890733; doi:10.1371/journal.pone.0296671)

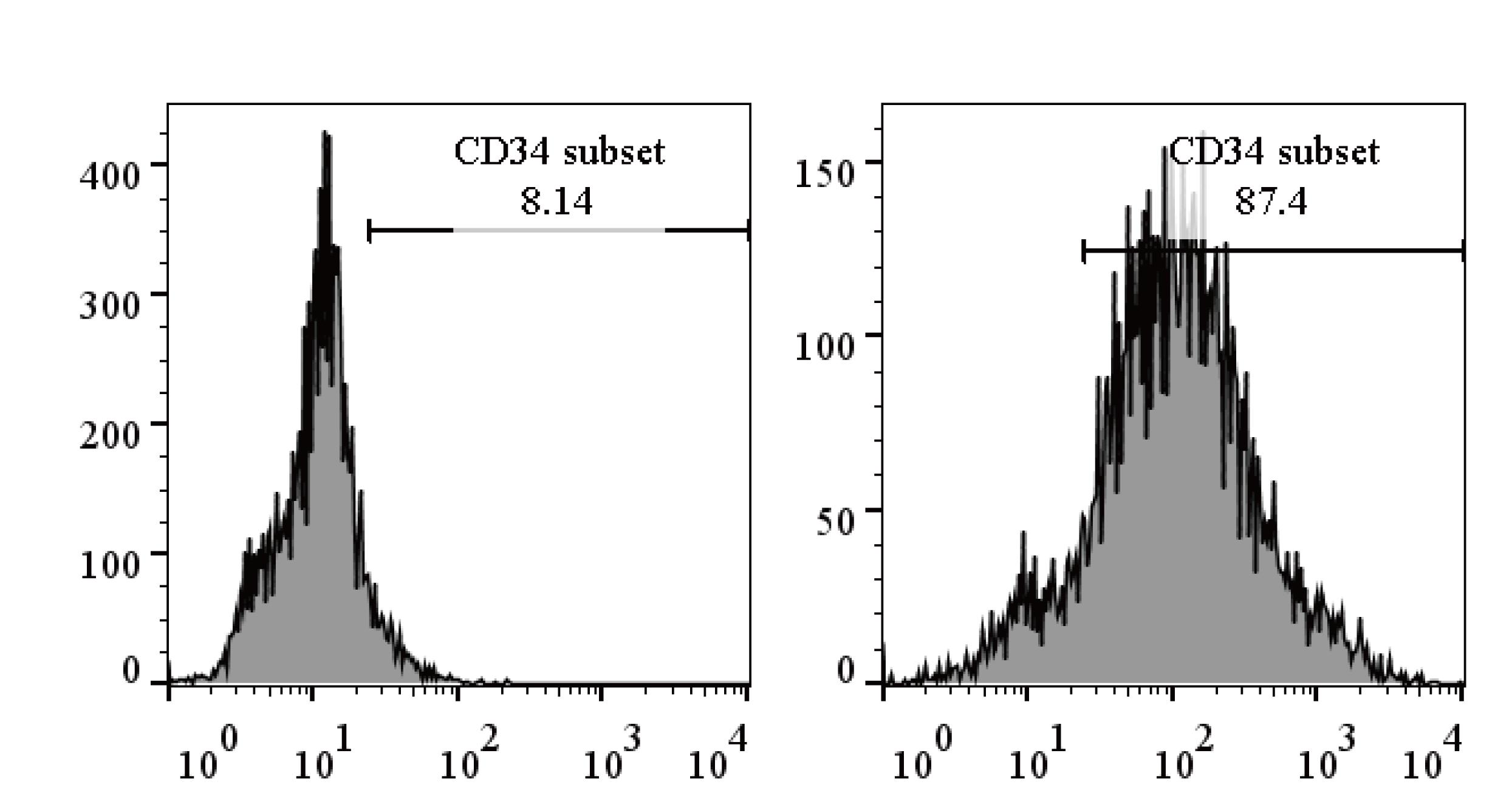

Supplement: S1 Raw data — (ZIP) [file pone.0296671.s002.zip › images/1C.tif]

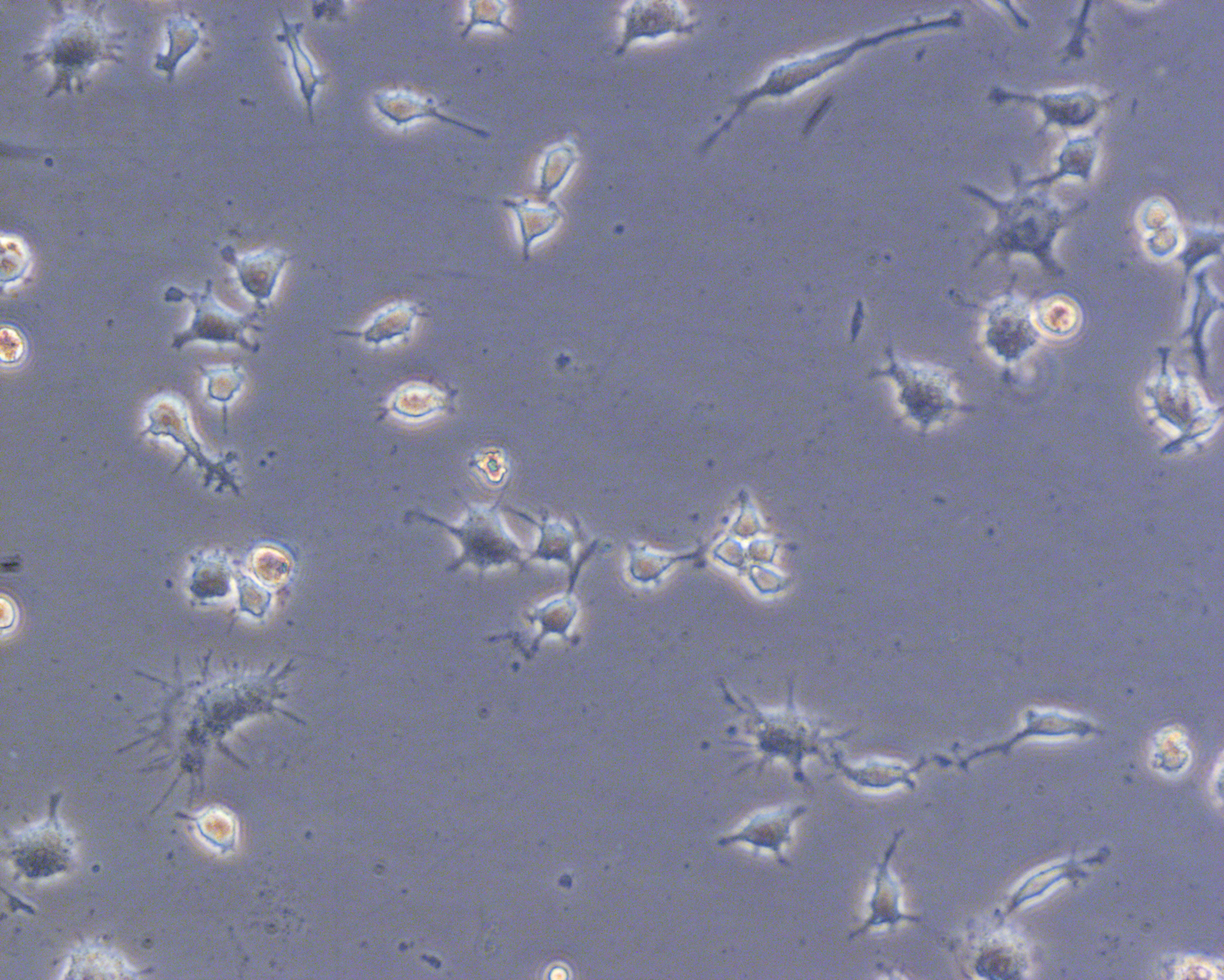

Supplement: S1 Raw data — (ZIP) [file pone.0296671.s002.zip › images/A/Day 3.tif]

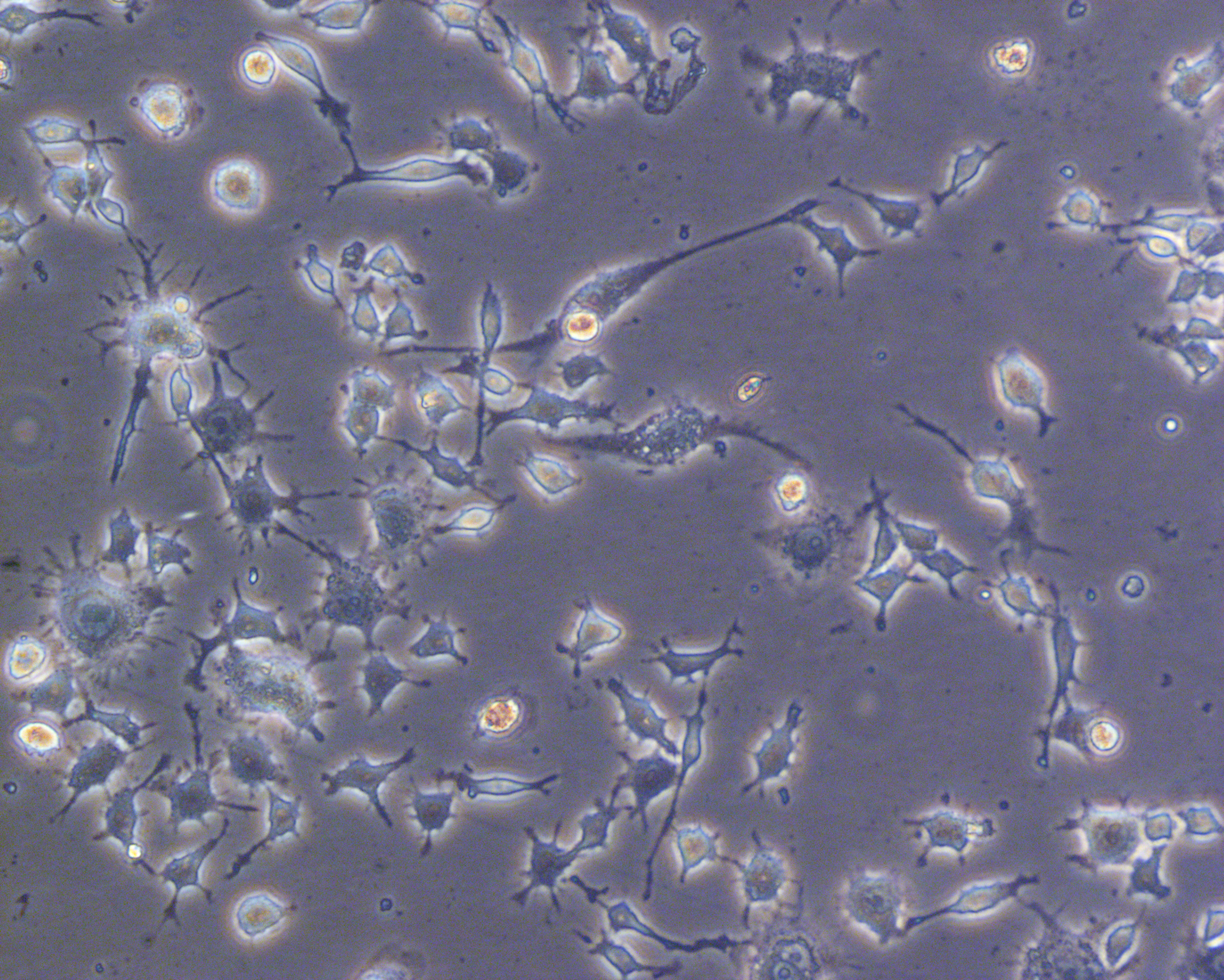

Supplement: S1 Raw data — (ZIP) [file pone.0296671.s002.zip › images/A/Day 7.tif]

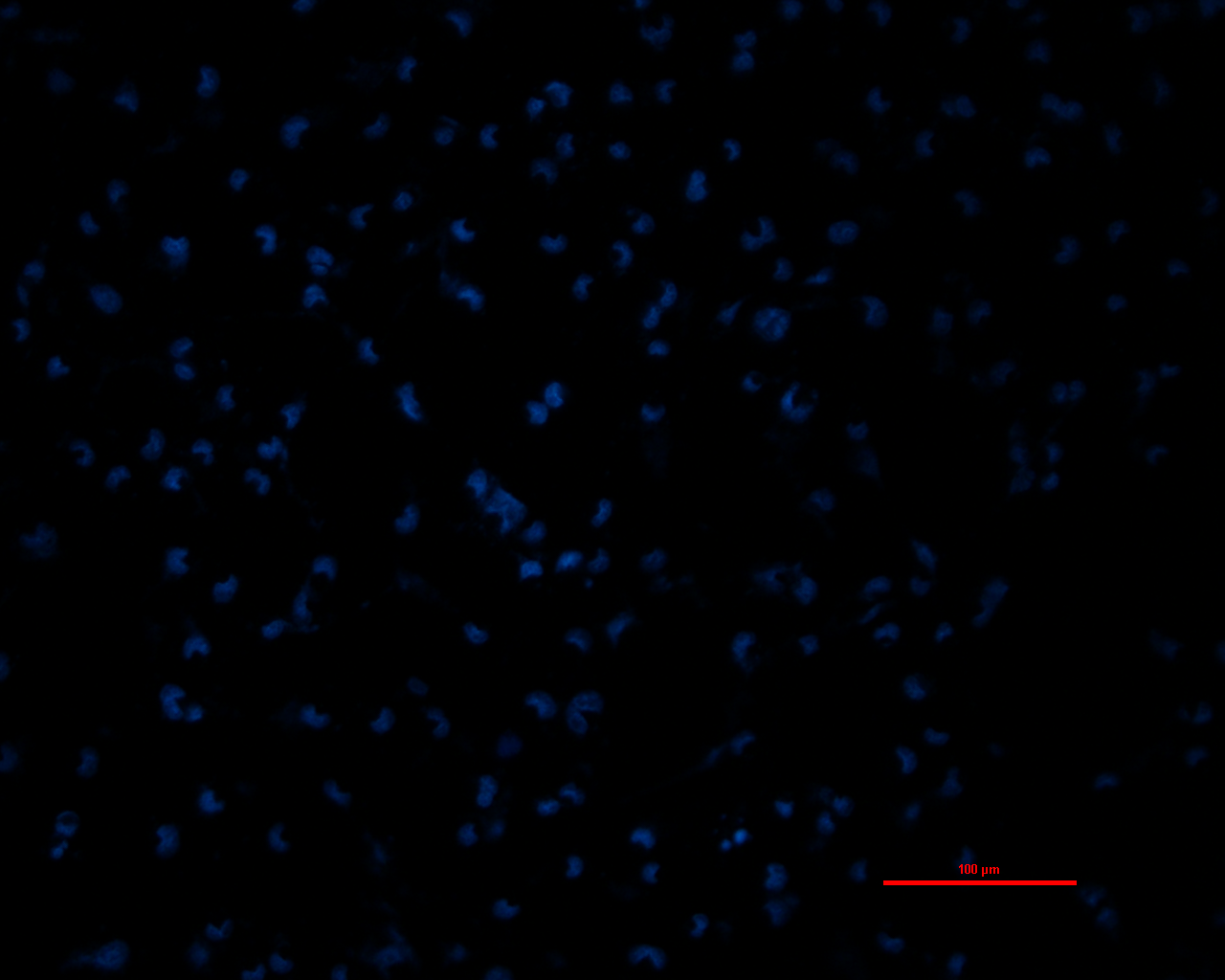

Supplement: S1 Raw data — (ZIP) [file pone.0296671.s002.zip › images/B/DAPI.tif]

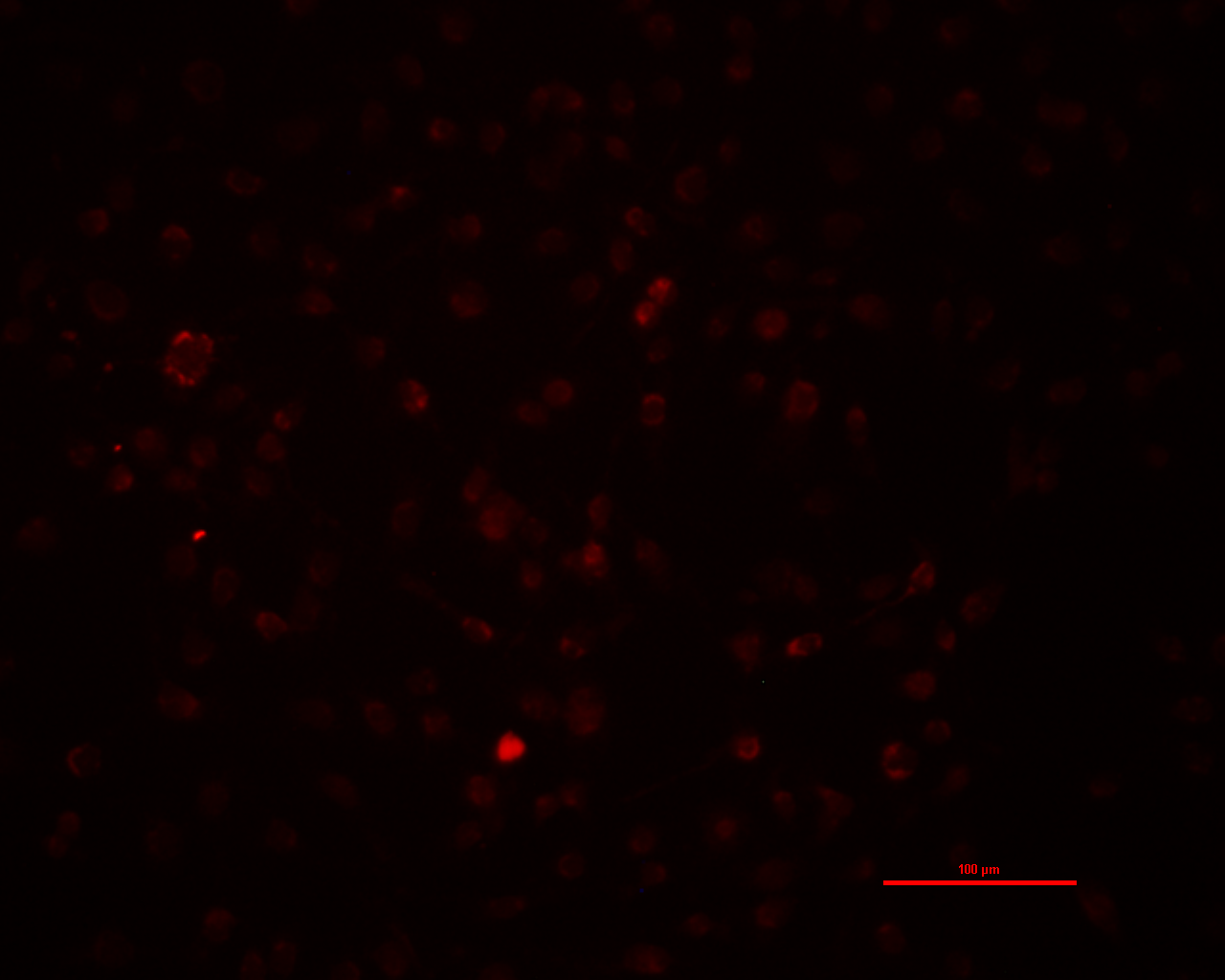

Supplement: S1 Raw data — (ZIP) [file pone.0296671.s002.zip › images/B/Dil-ac-LDL.tif]

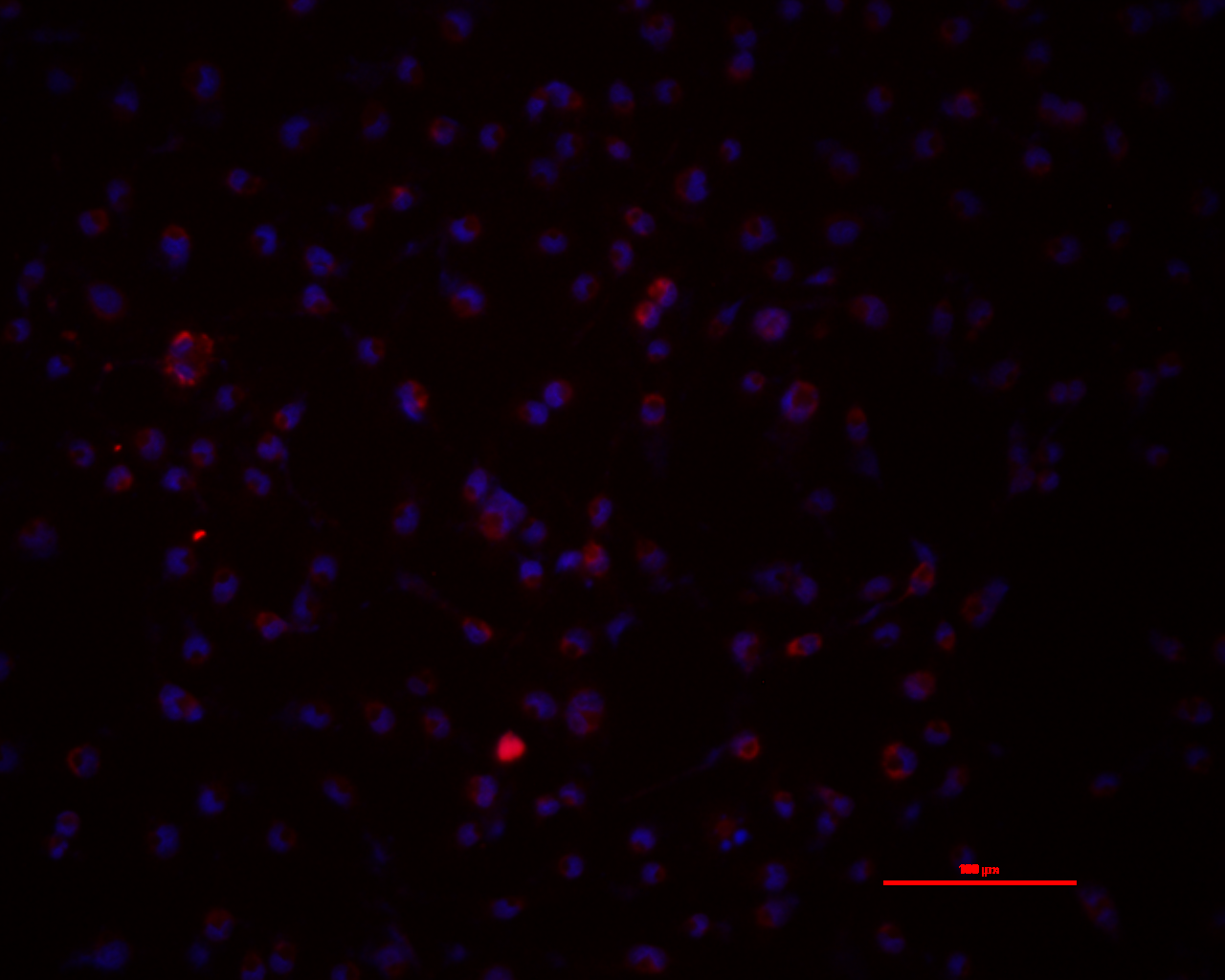

Supplement: S1 Raw data — (ZIP) [file pone.0296671.s002.zip › images/B/Merge.tif]

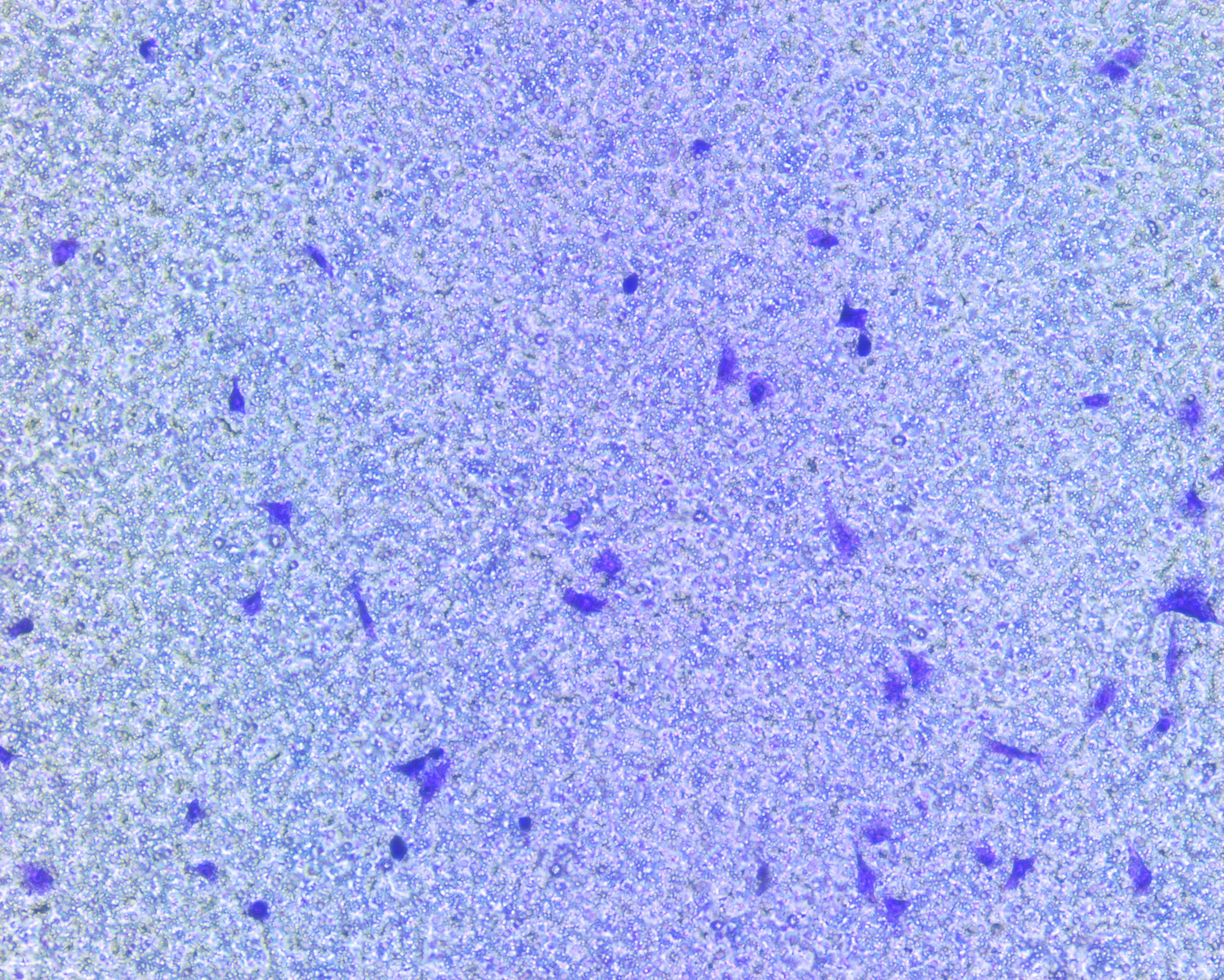

Supplement: S2 Raw data — (ZIP) [file pone.0296671.s003.zip › images/2D/miR-155 inhibitor.tif]

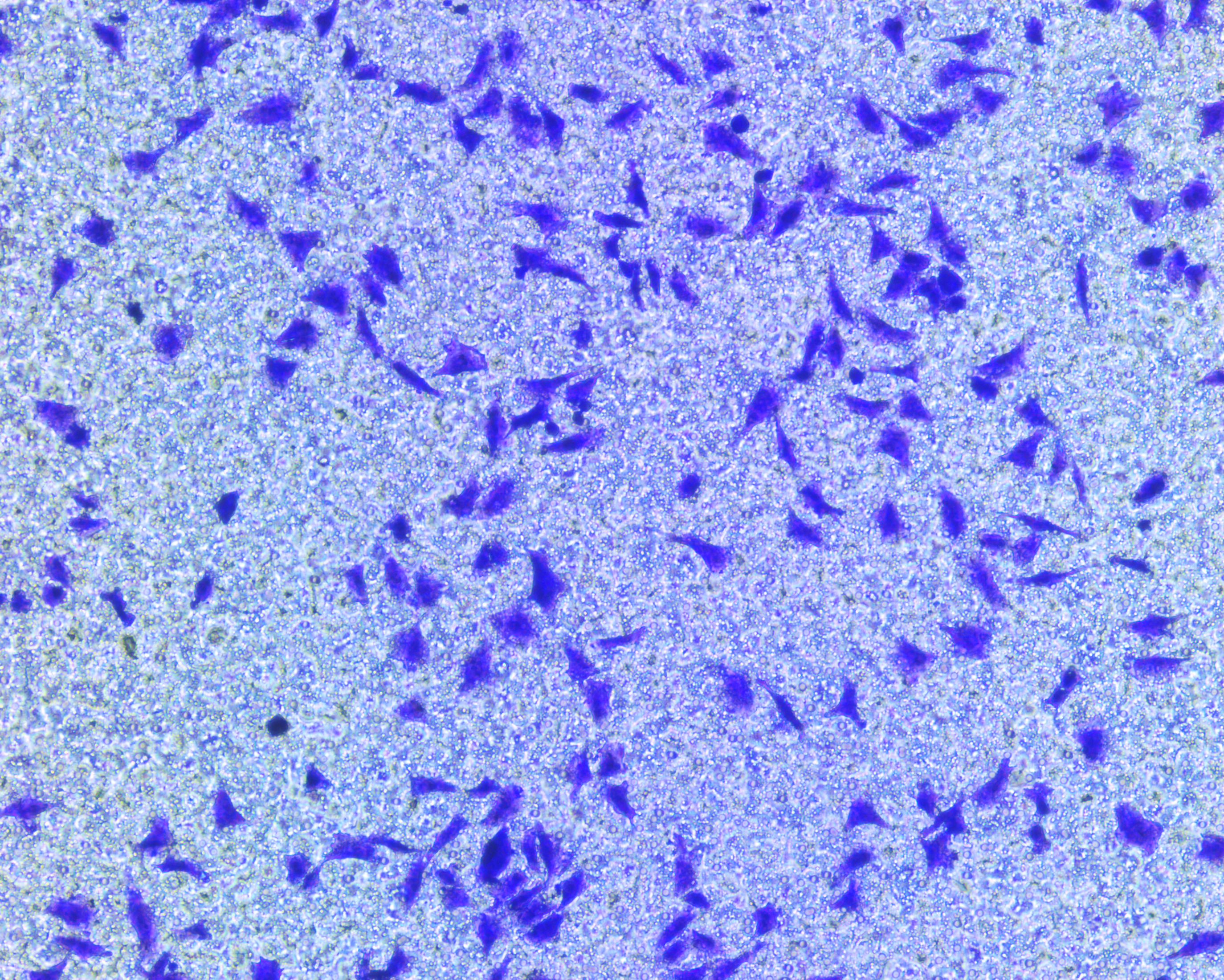

Supplement: S2 Raw data — (ZIP) [file pone.0296671.s003.zip › images/2D/miR-155 mimics.tif]

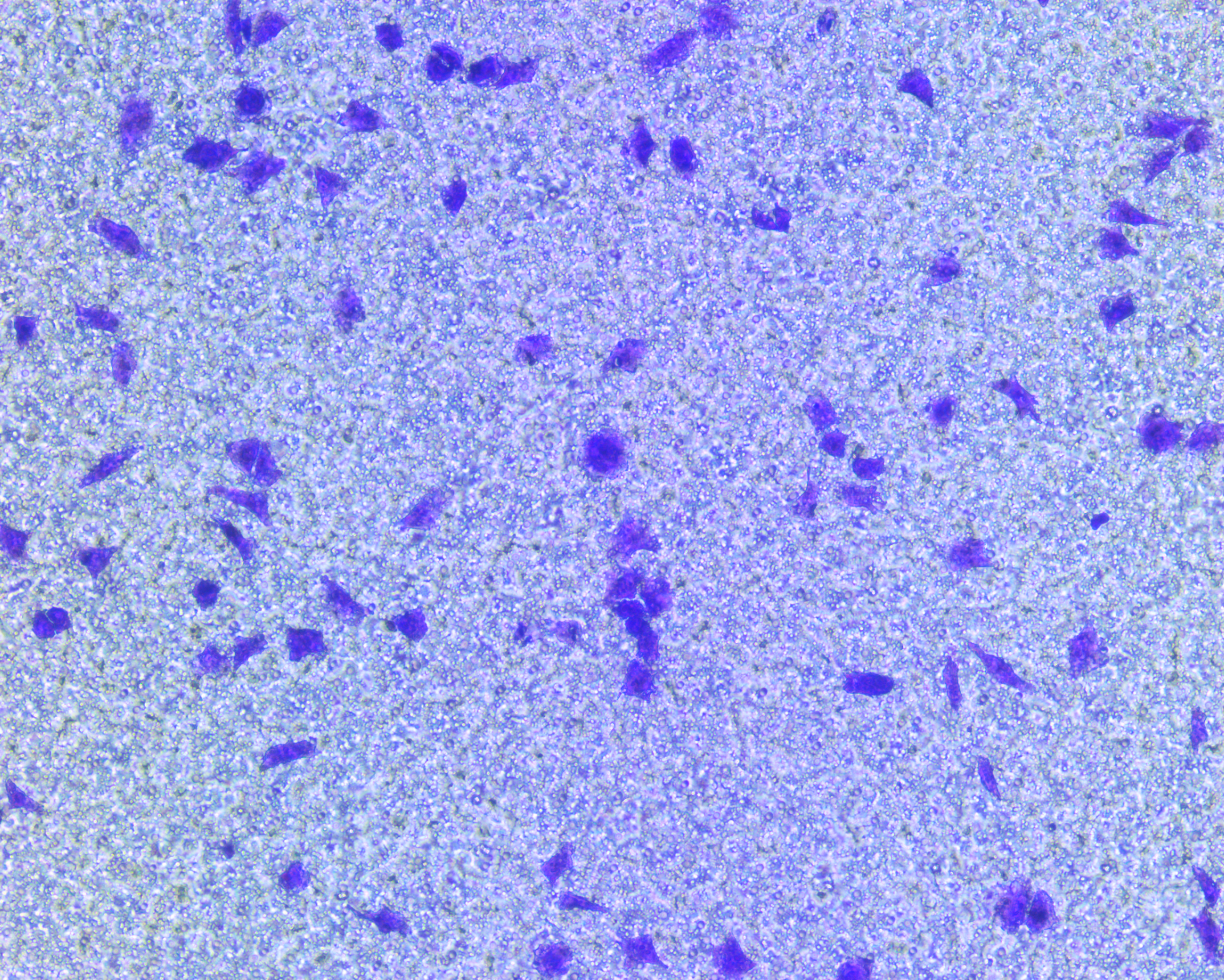

Supplement: S2 Raw data — (ZIP) [file pone.0296671.s003.zip › images/2D/NC.tif]

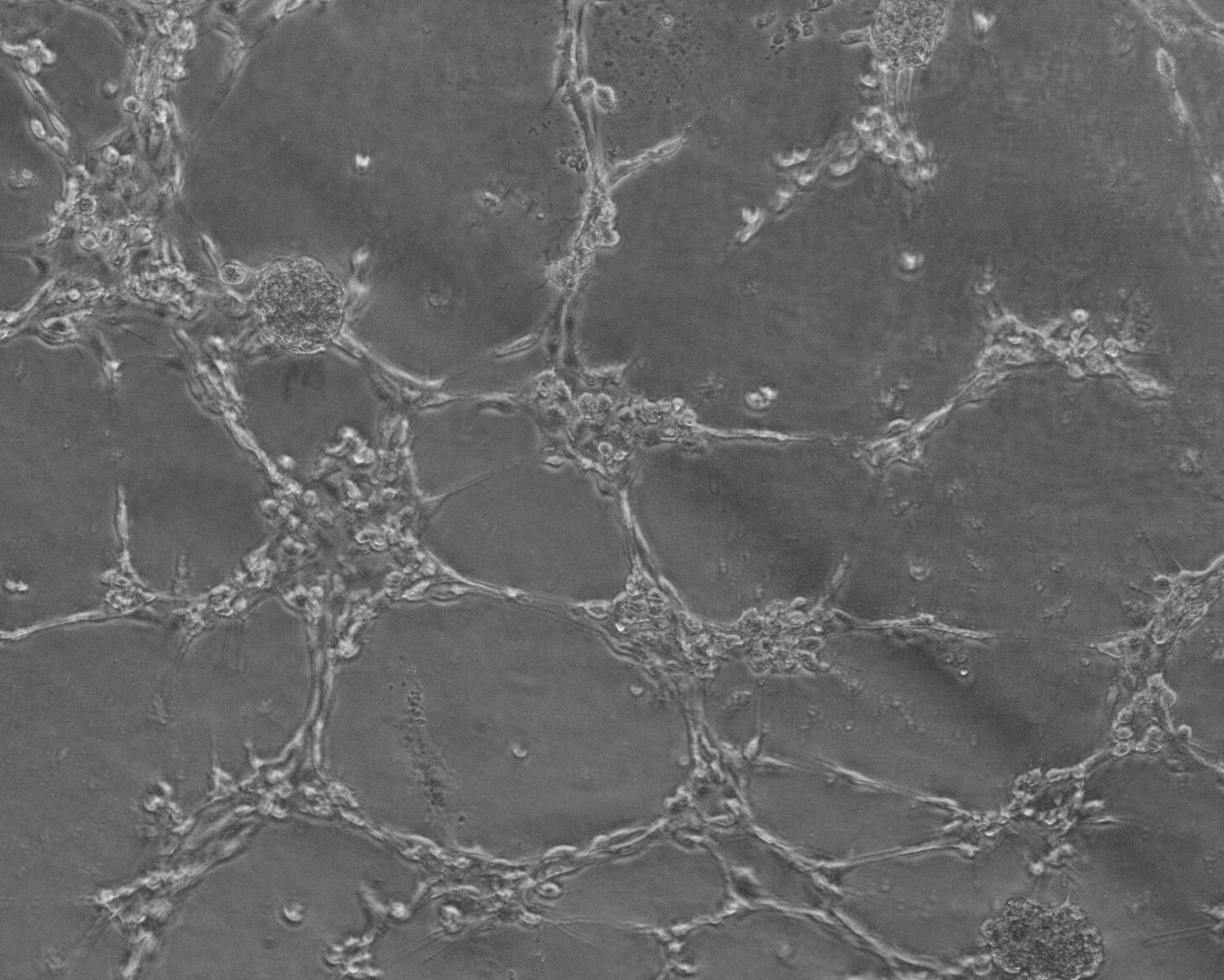

Supplement: S2 Raw data — (ZIP) [file pone.0296671.s003.zip › images/2E/miR-155inhibitor.tif]

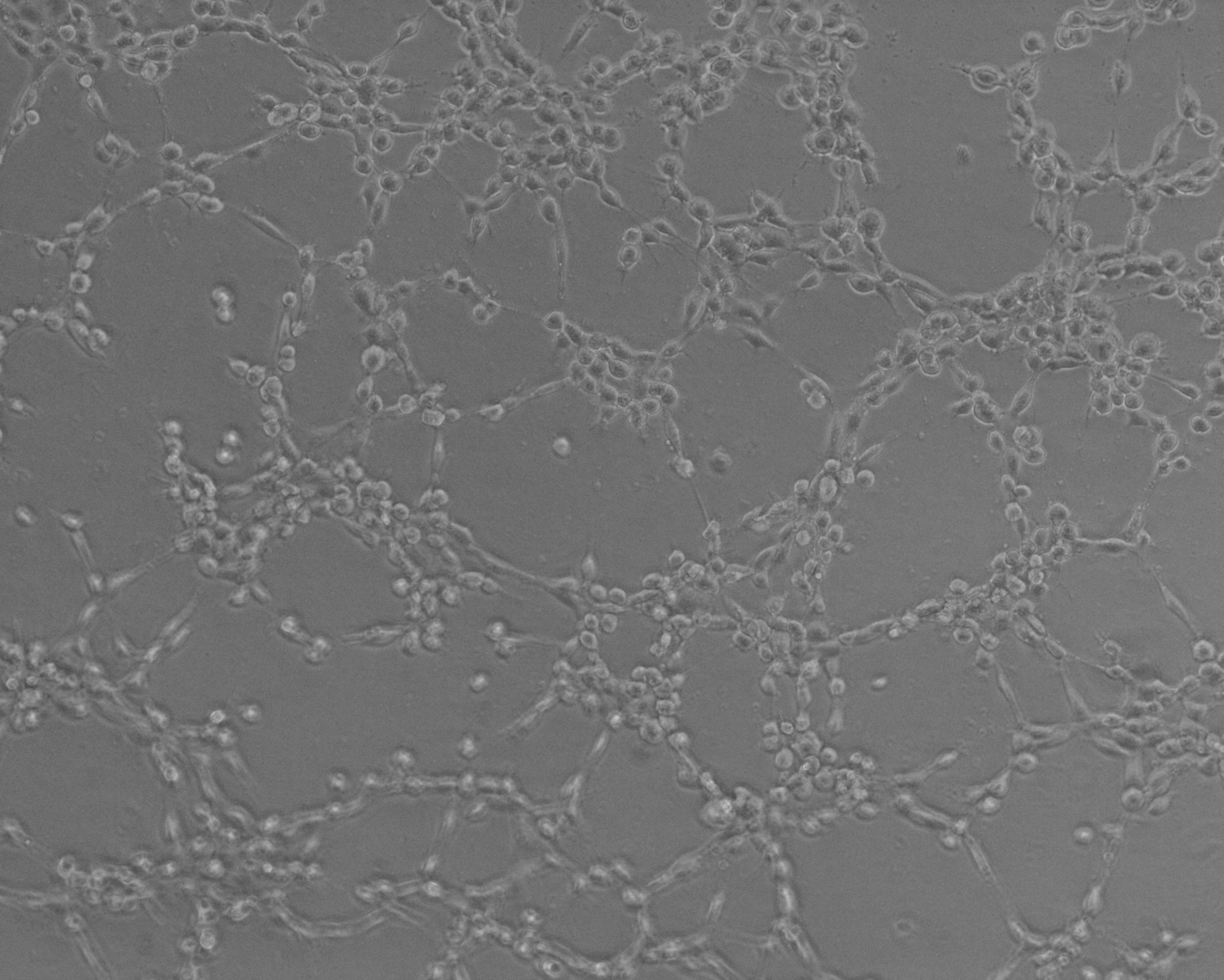

Supplement: S2 Raw data — (ZIP) [file pone.0296671.s003.zip › images/2E/miR-155mimics.tif]

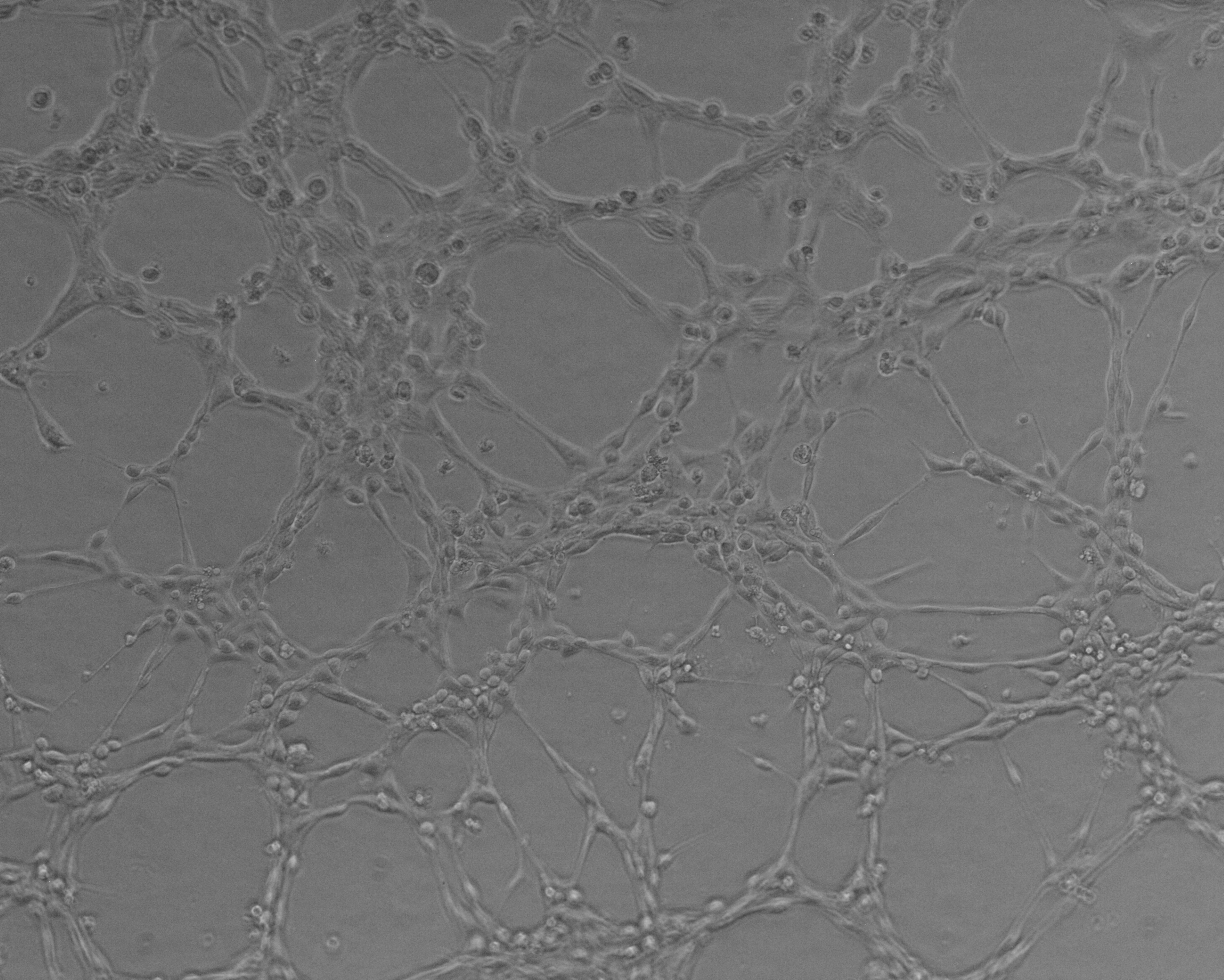

Supplement: S2 Raw data — (ZIP) [file pone.0296671.s003.zip › images/2E/NC.tif]

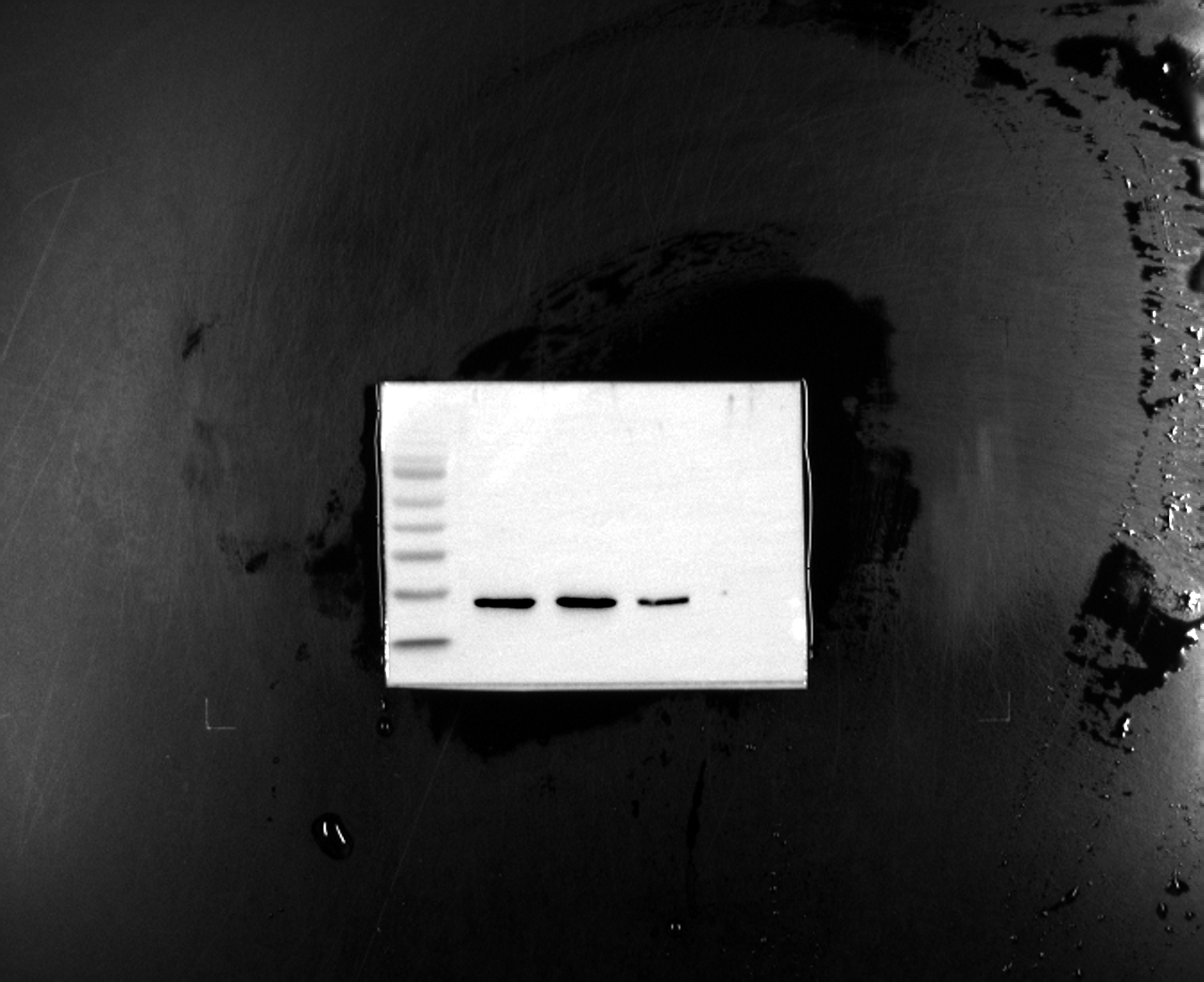

Supplement: S2 Raw data — (ZIP) [file pone.0296671.s003.zip › images/2G/1 VEGF.tif]

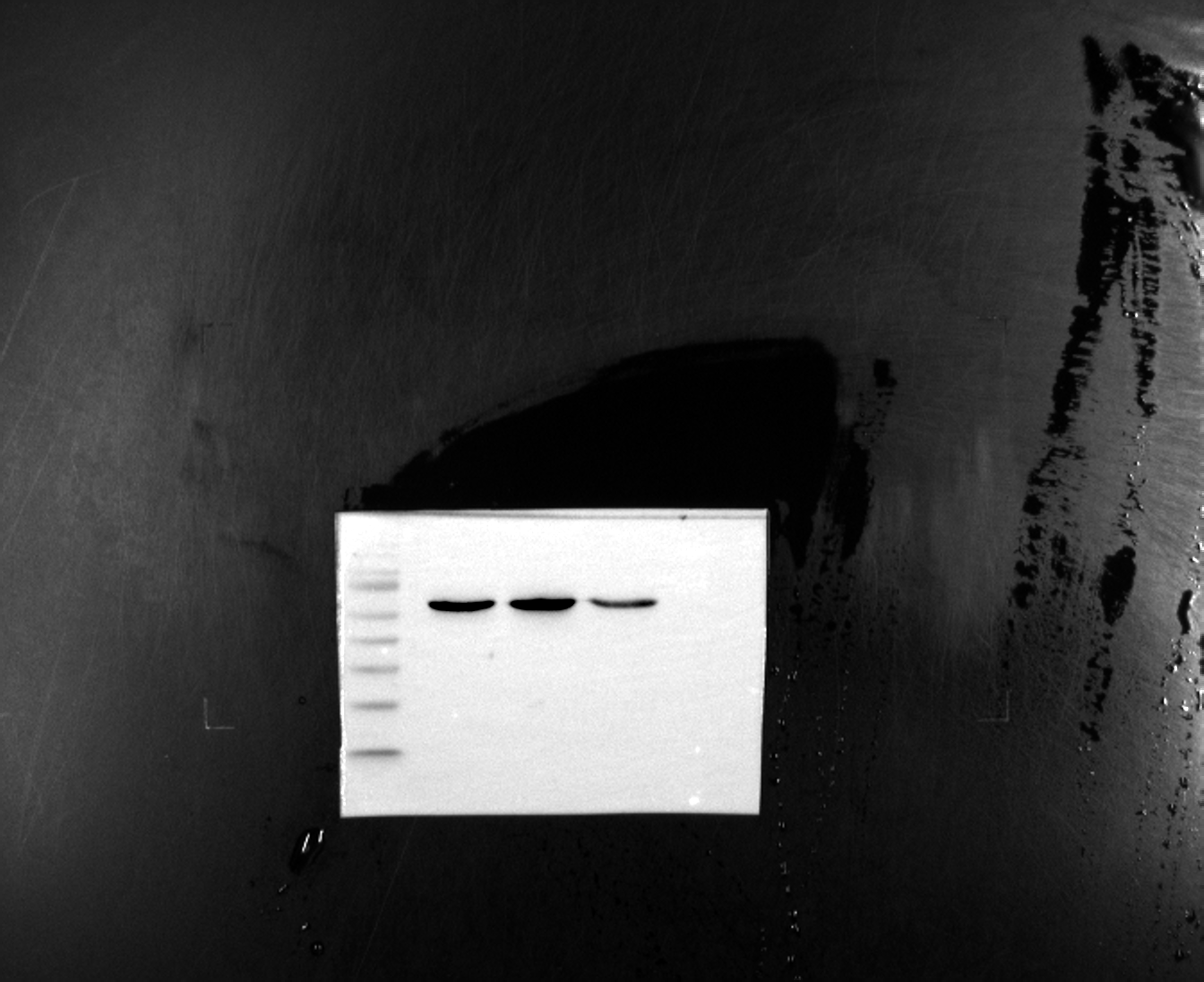

Supplement: S2 Raw data — (ZIP) [file pone.0296671.s003.zip › images/2G/2 MMP-2.tif]

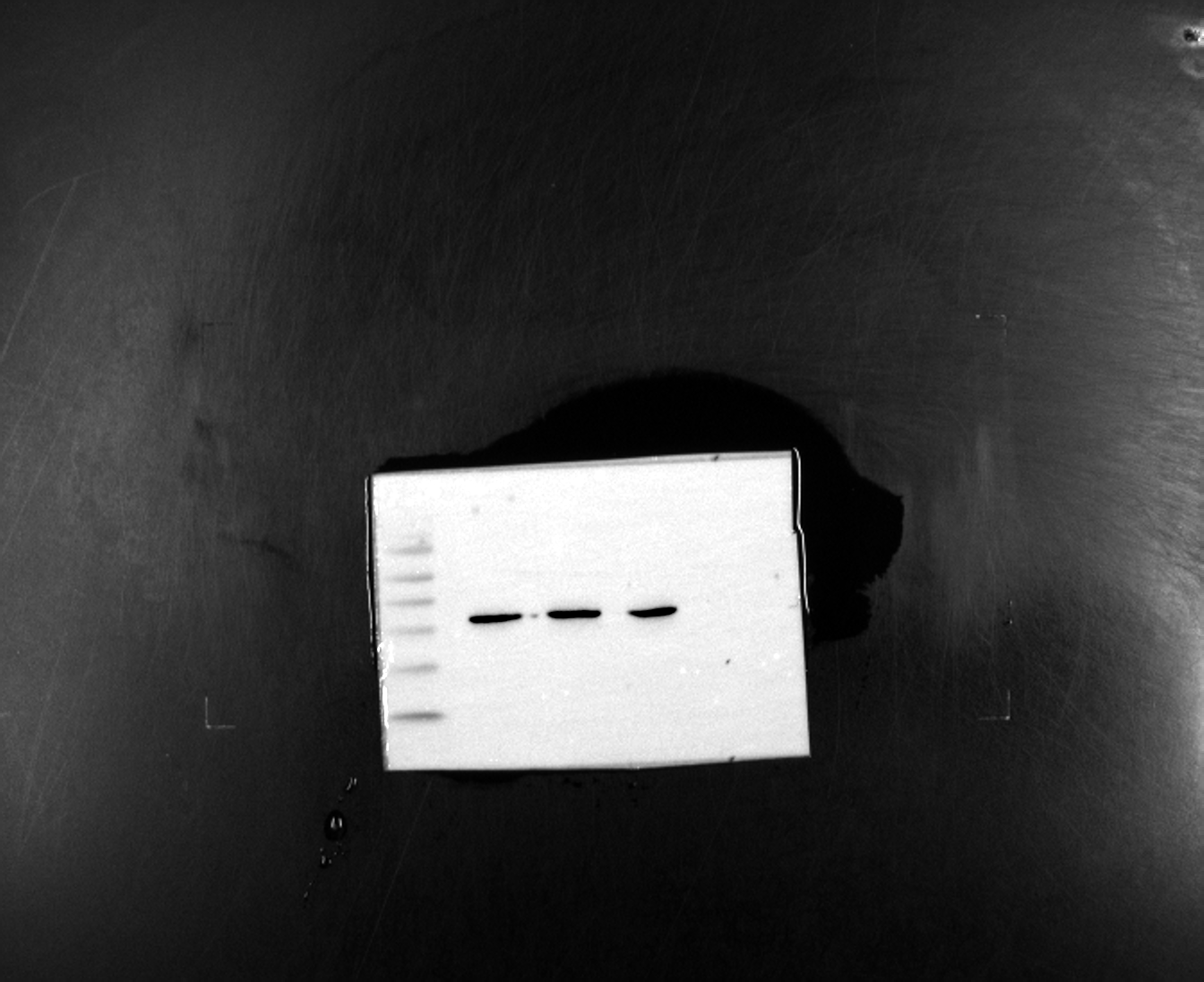

Supplement: S2 Raw data — (ZIP) [file pone.0296671.s003.zip › images/2G/3 GAPDH.tif]

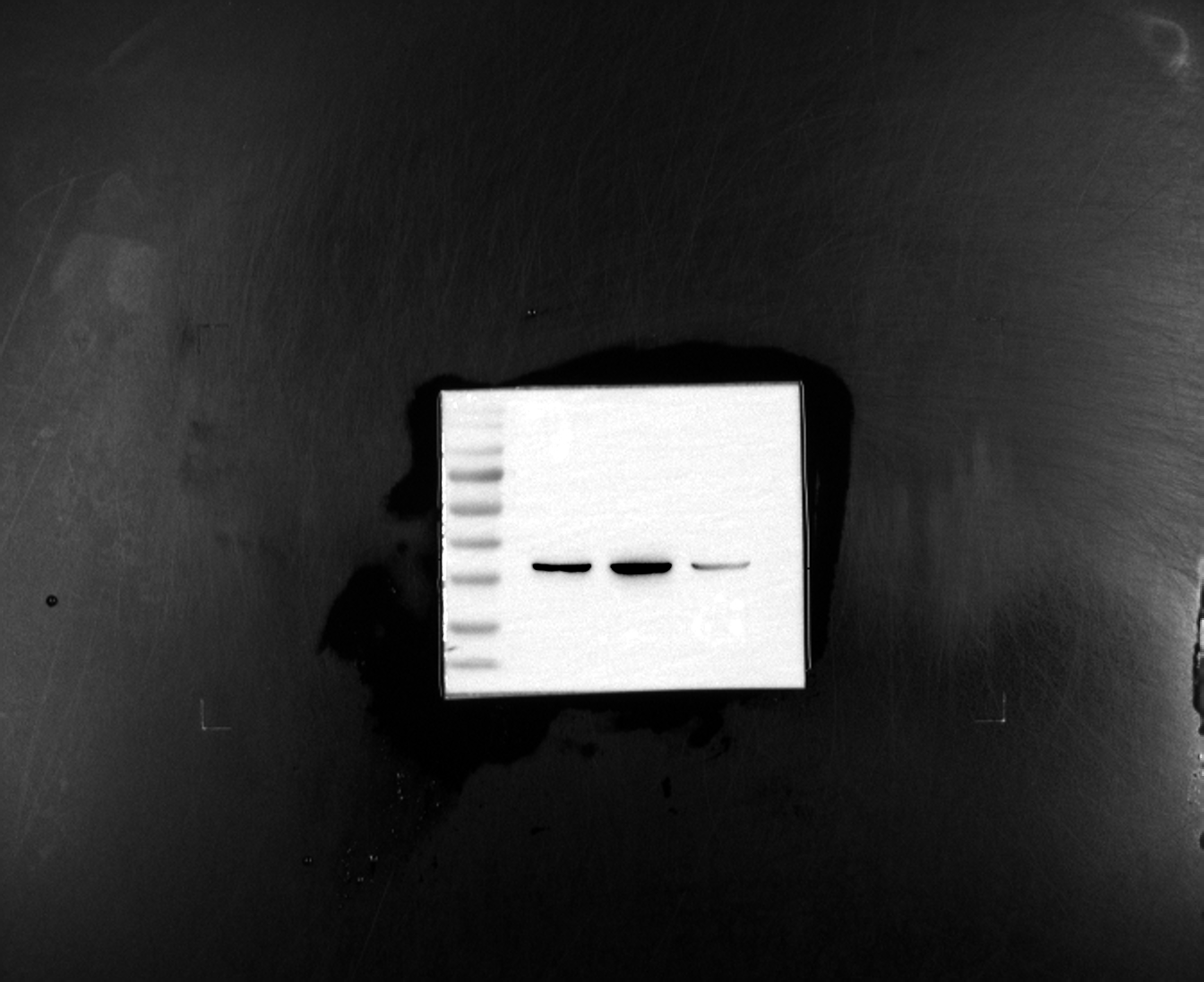

Supplement: S3 Raw data — (ZIP) [file pone.0296671.s004.zip › images/3A/1 JAPID2.tif]

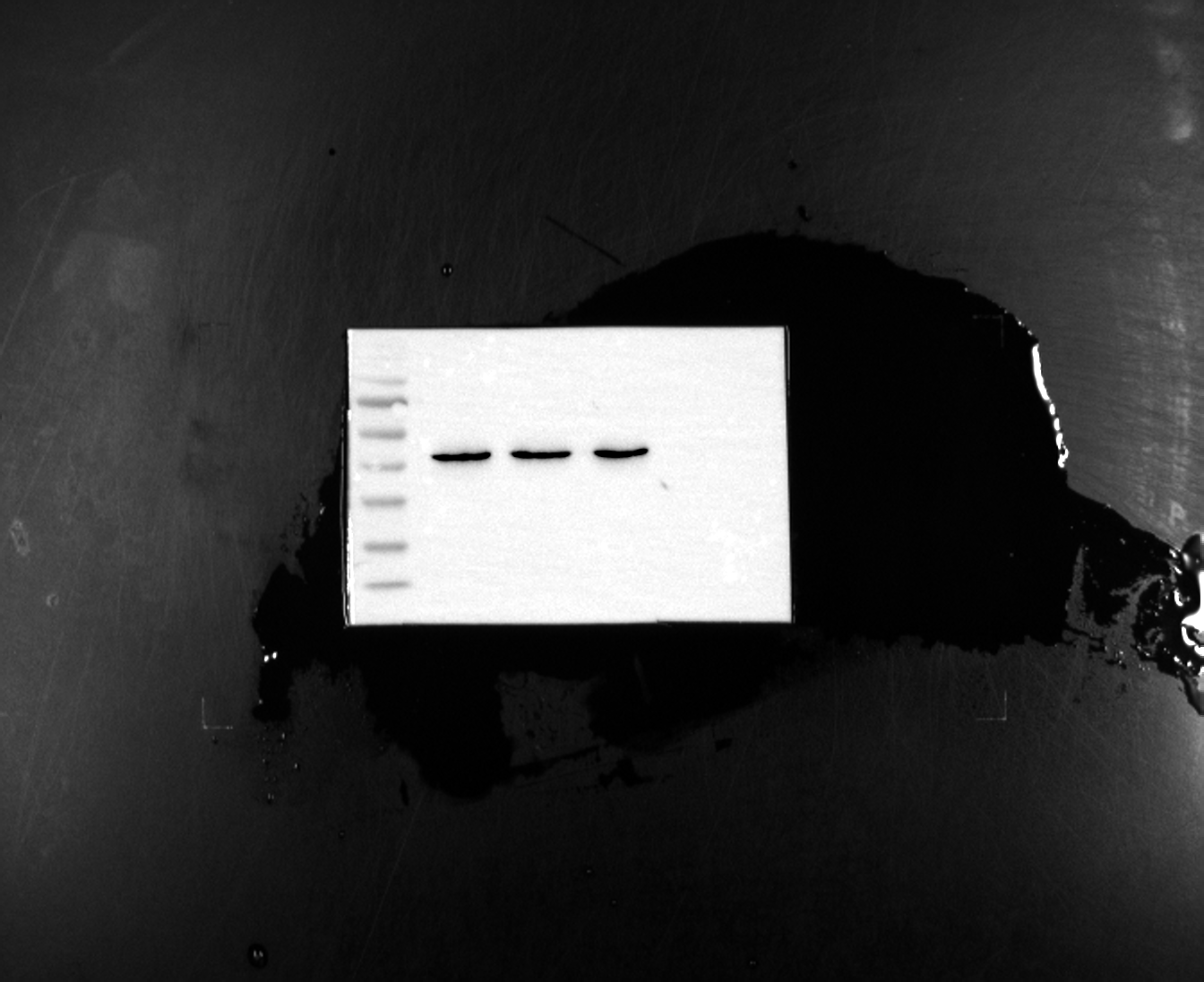

Supplement: S3 Raw data — (ZIP) [file pone.0296671.s004.zip › images/3A/2 β-actin.tif]

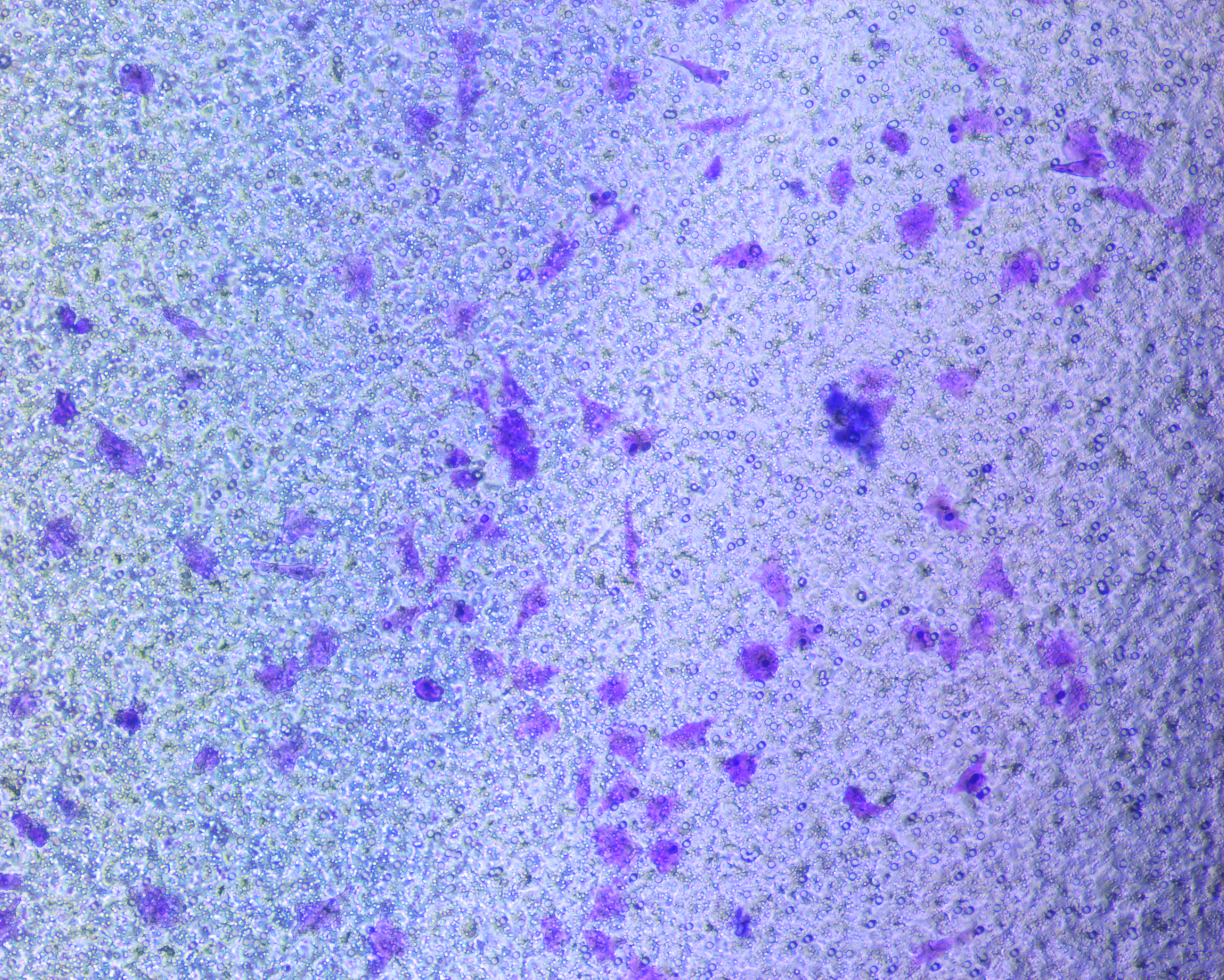

Supplement: S3 Raw data — (ZIP) [file pone.0296671.s004.zip › images/3D/NC.tif]

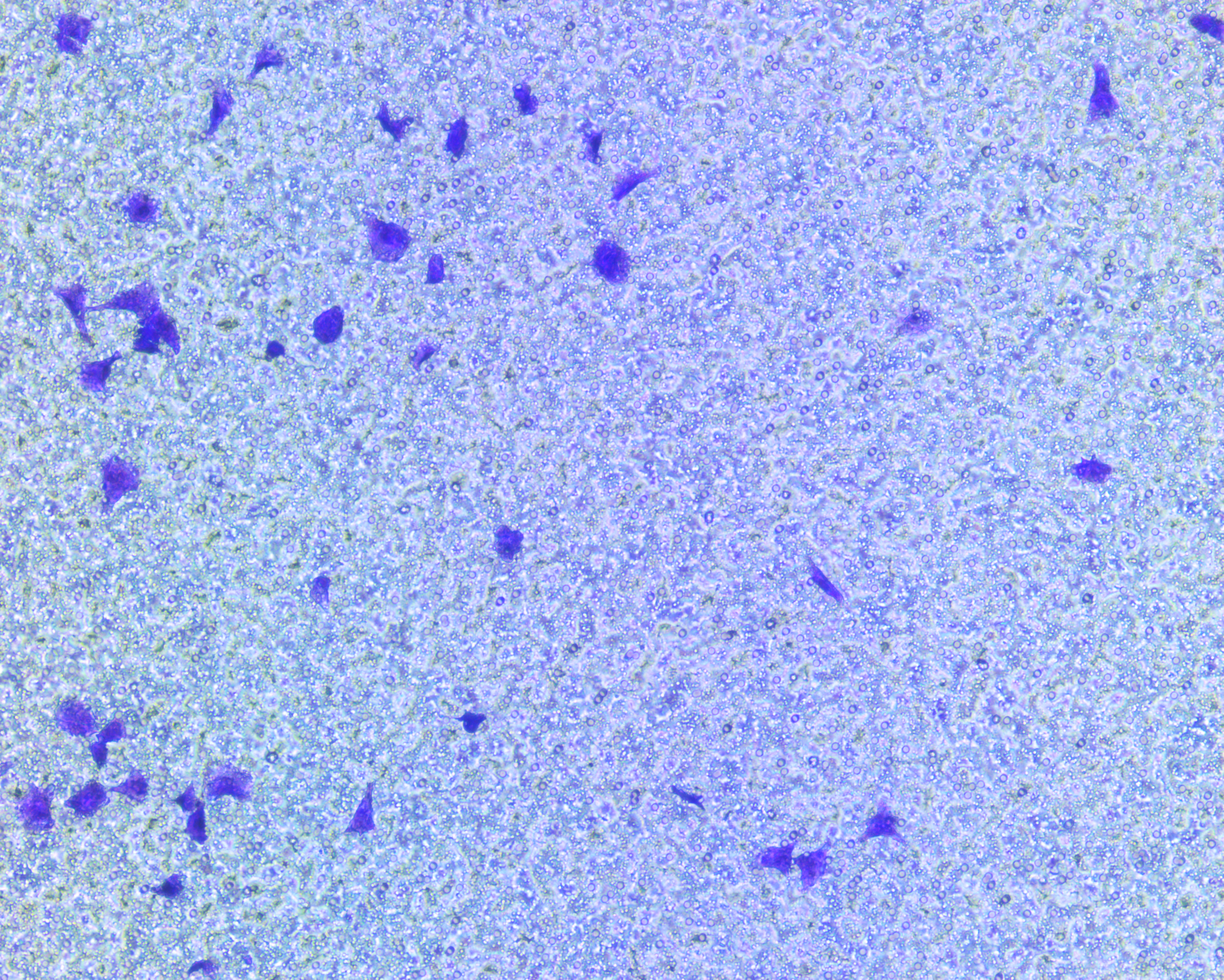

Supplement: S3 Raw data — (ZIP) [file pone.0296671.s004.zip › images/3D/pc-JARID2.tif]

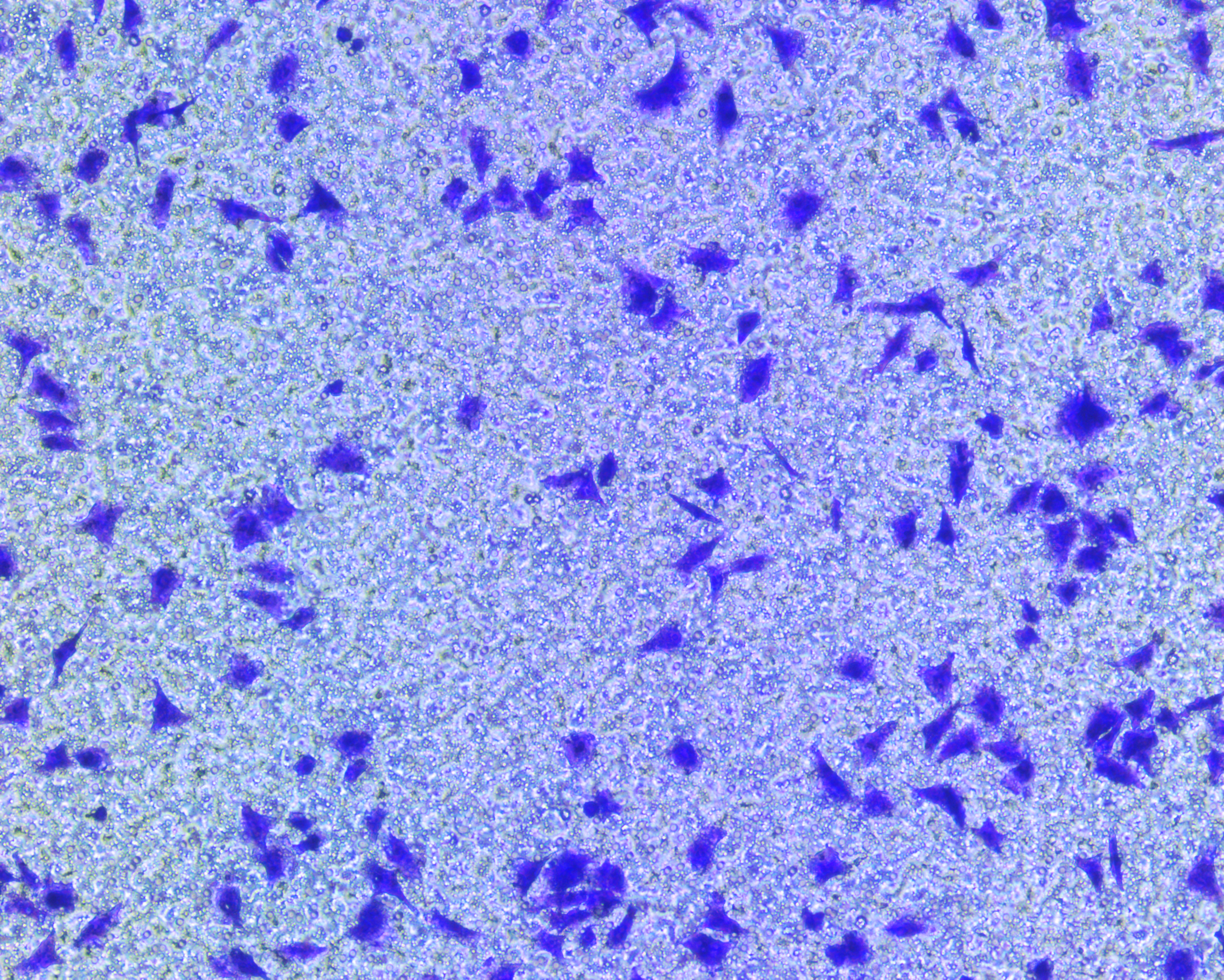

Supplement: S3 Raw data — (ZIP) [file pone.0296671.s004.zip › images/3D/si-JARID2.tif]

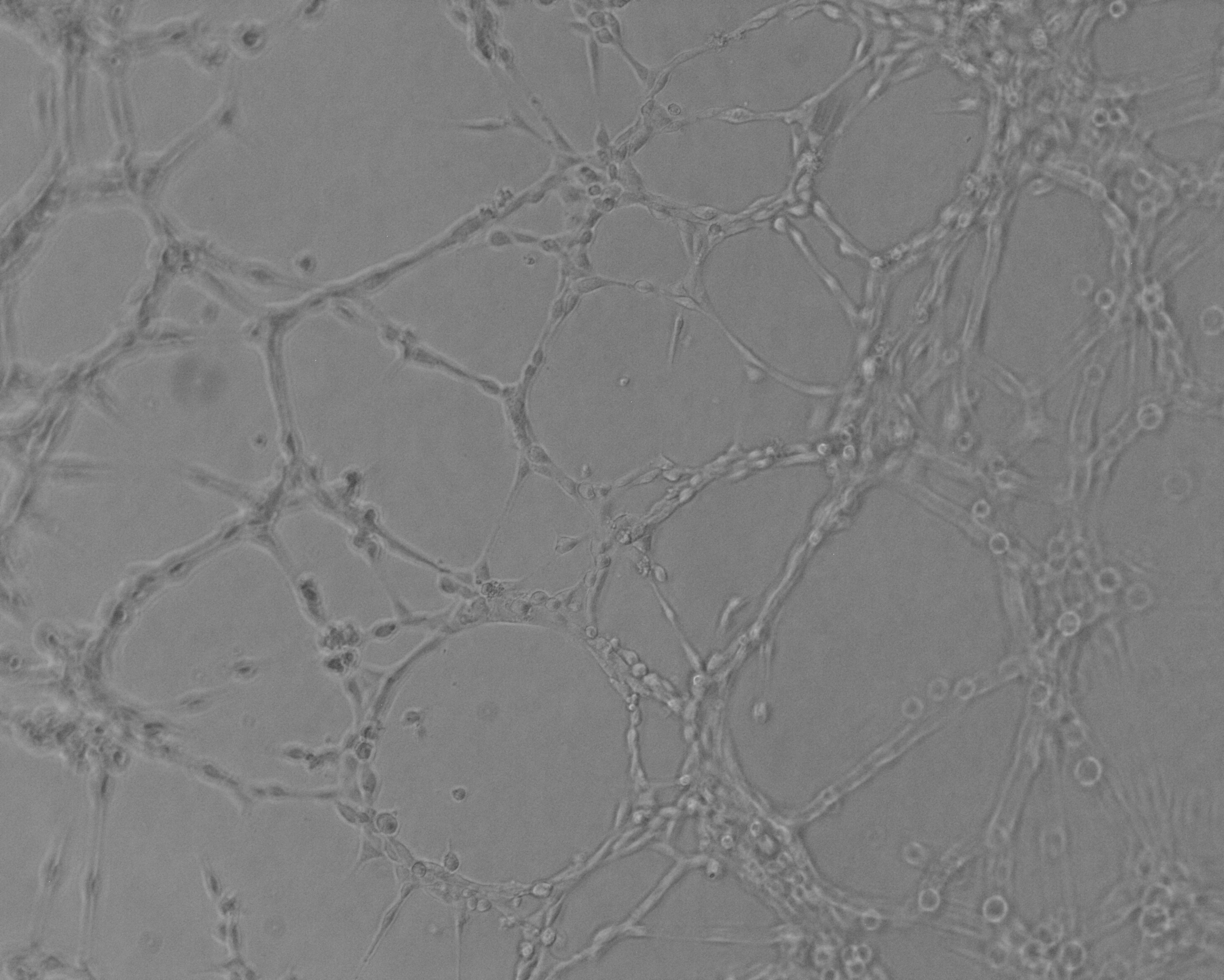

Supplement: S3 Raw data — (ZIP) [file pone.0296671.s004.zip › images/3E/NC.tif]

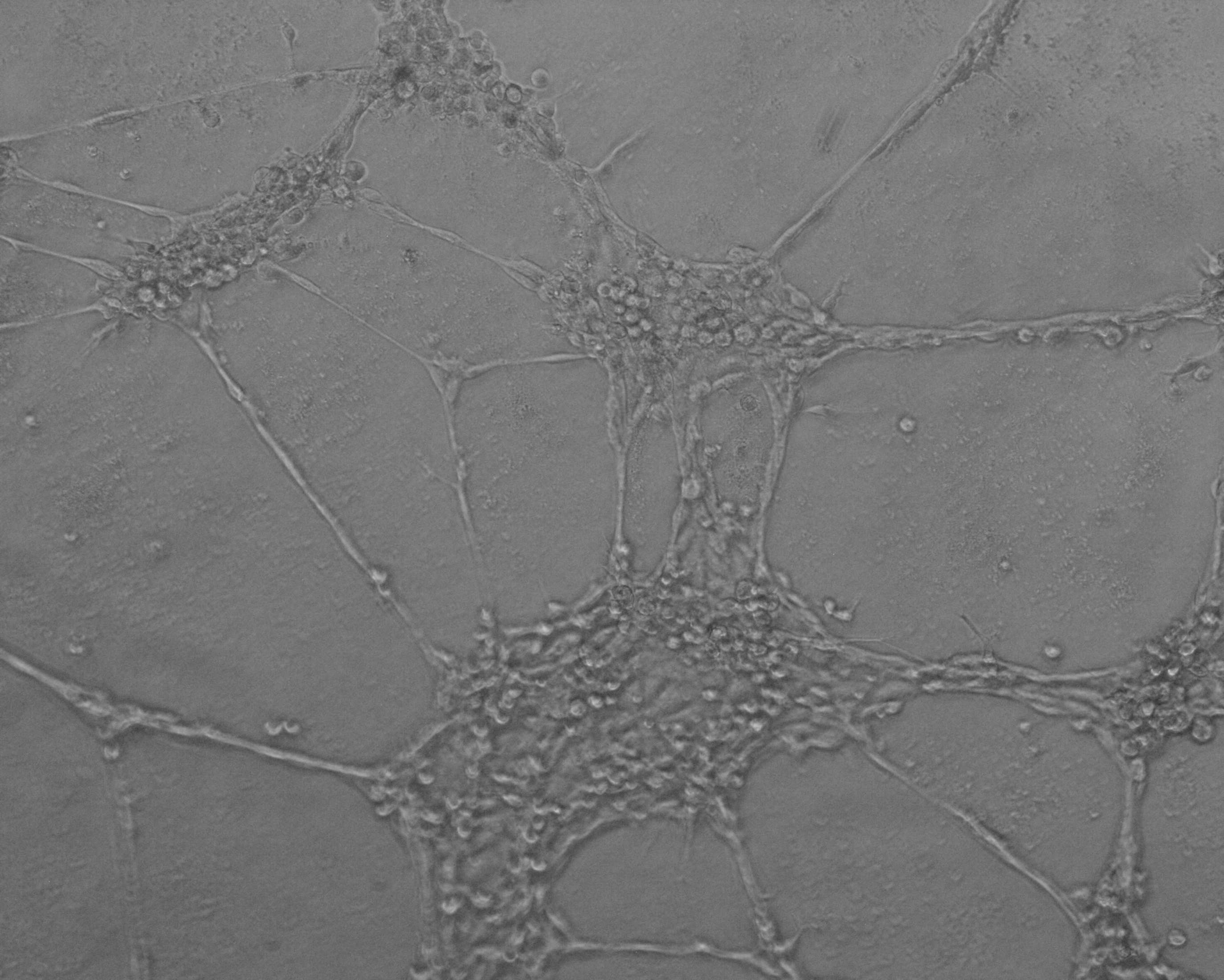

Supplement: S3 Raw data — (ZIP) [file pone.0296671.s004.zip › images/3E/pc-JARID2.tif]

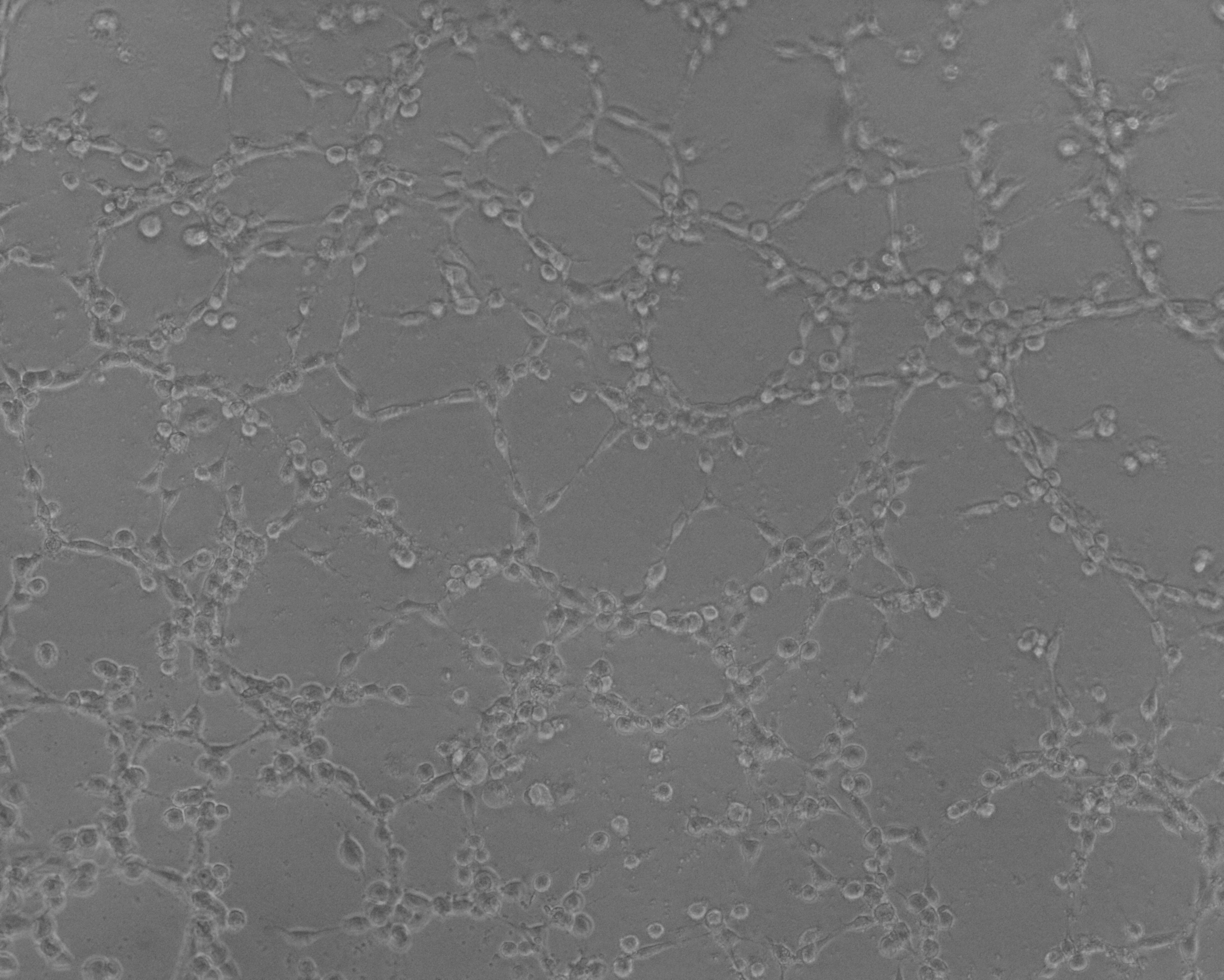

Supplement: S3 Raw data — (ZIP) [file pone.0296671.s004.zip › images/3E/si-JARID2.tif]

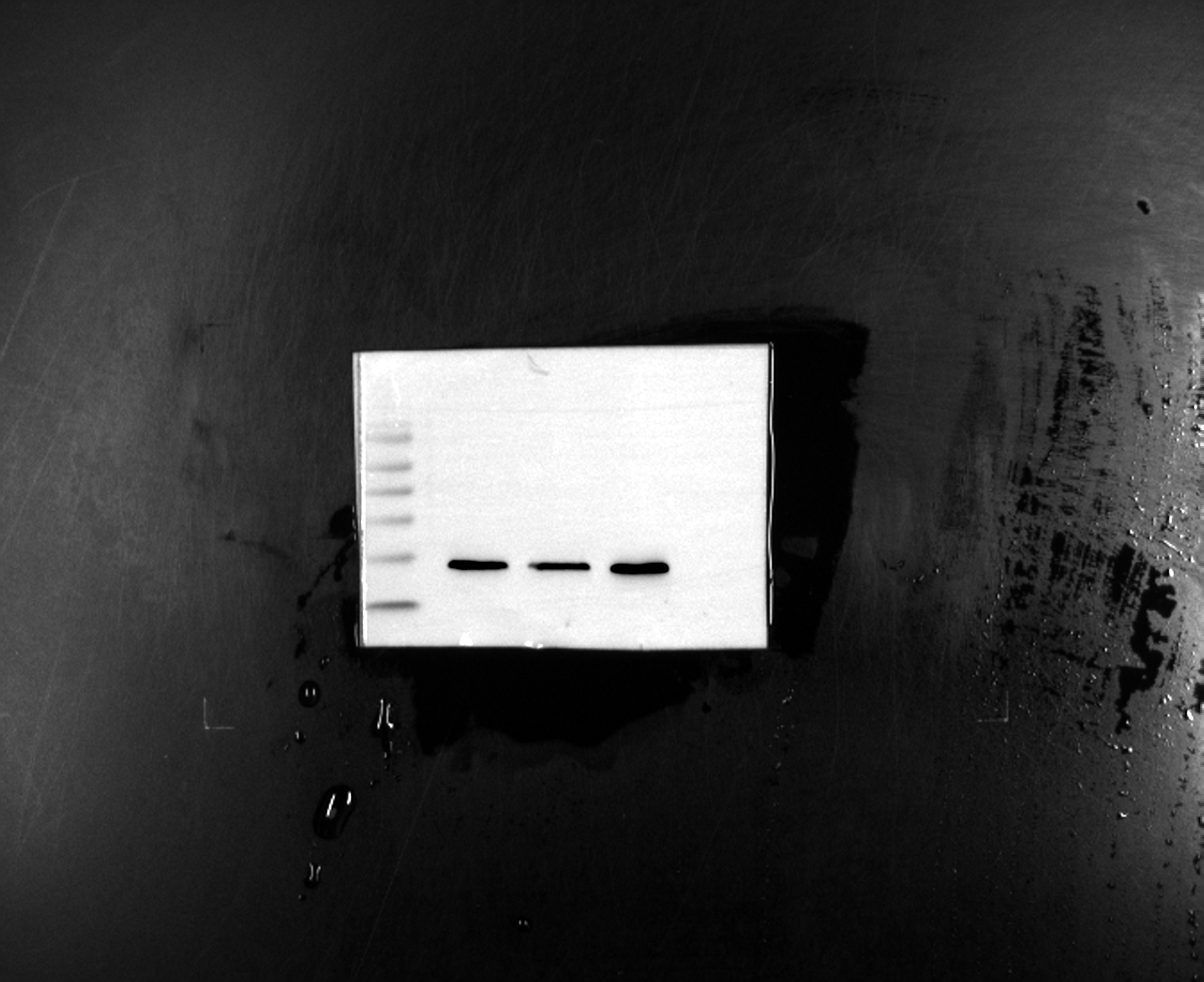

Supplement: S3 Raw data — (ZIP) [file pone.0296671.s004.zip › images/3G/1 VEGF.tif]

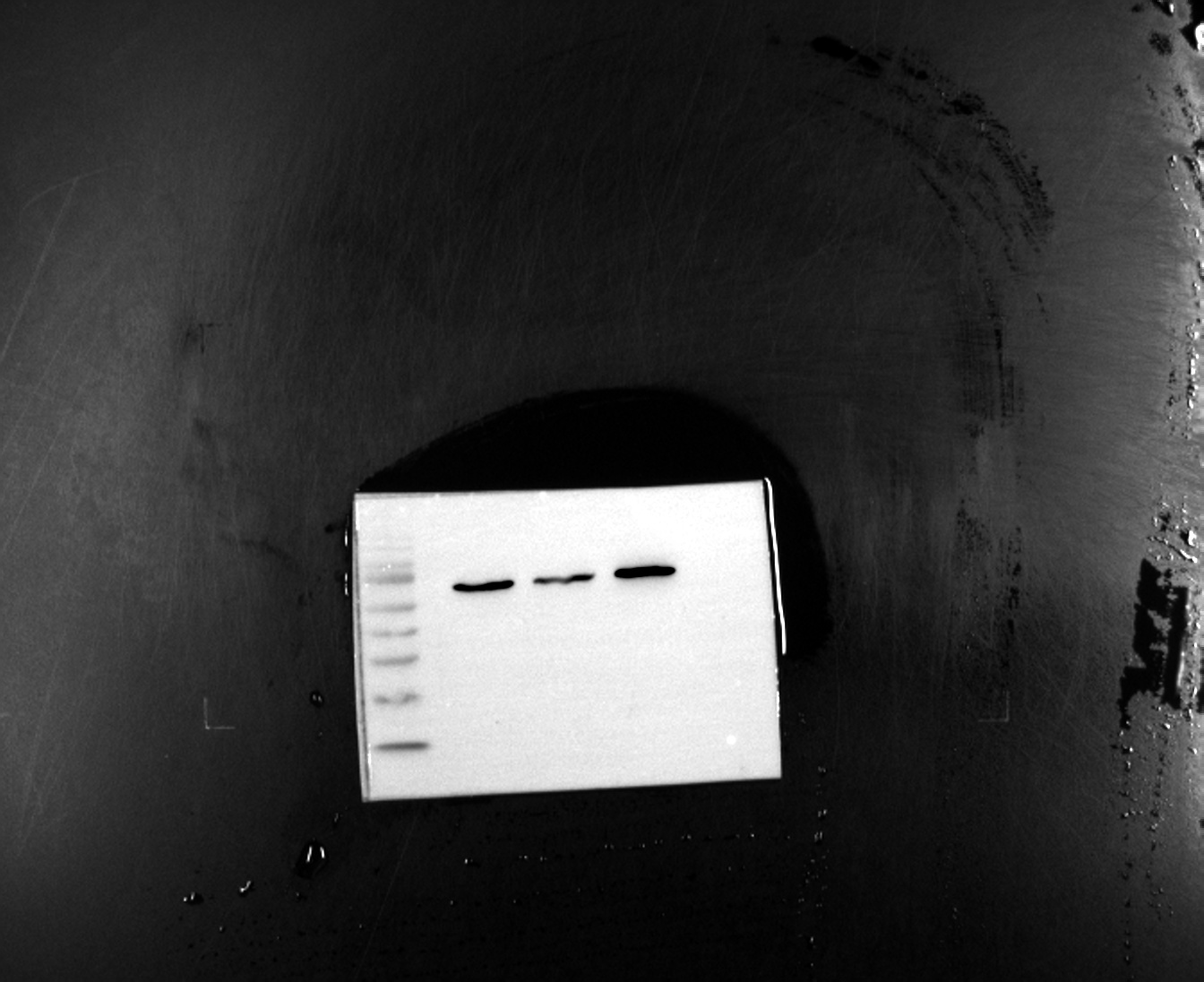

Supplement: S3 Raw data — (ZIP) [file pone.0296671.s004.zip › images/3G/2 MMP-2.tif]

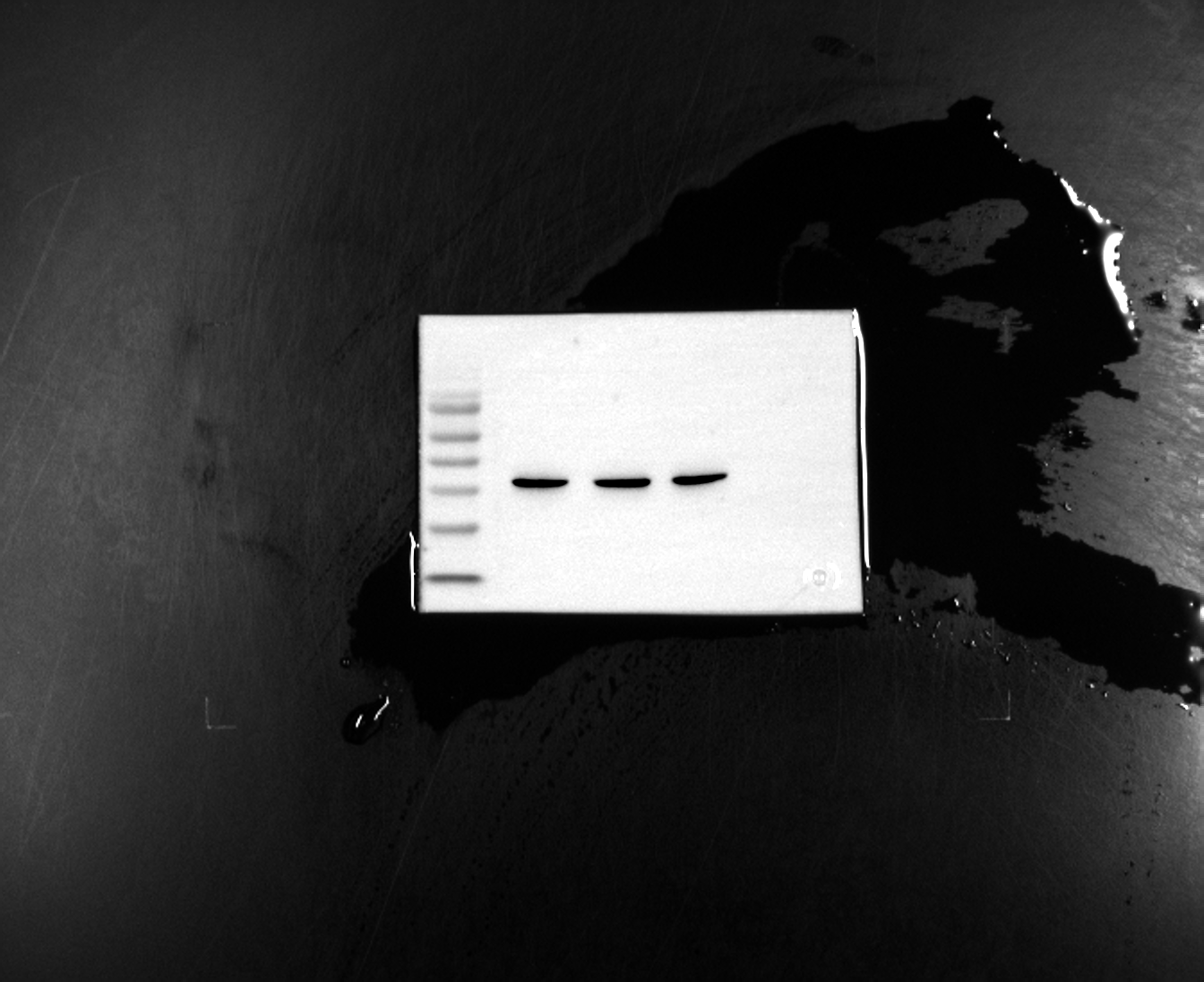

Supplement: S3 Raw data — (ZIP) [file pone.0296671.s004.zip › images/3G/3 GAPDH.tif]

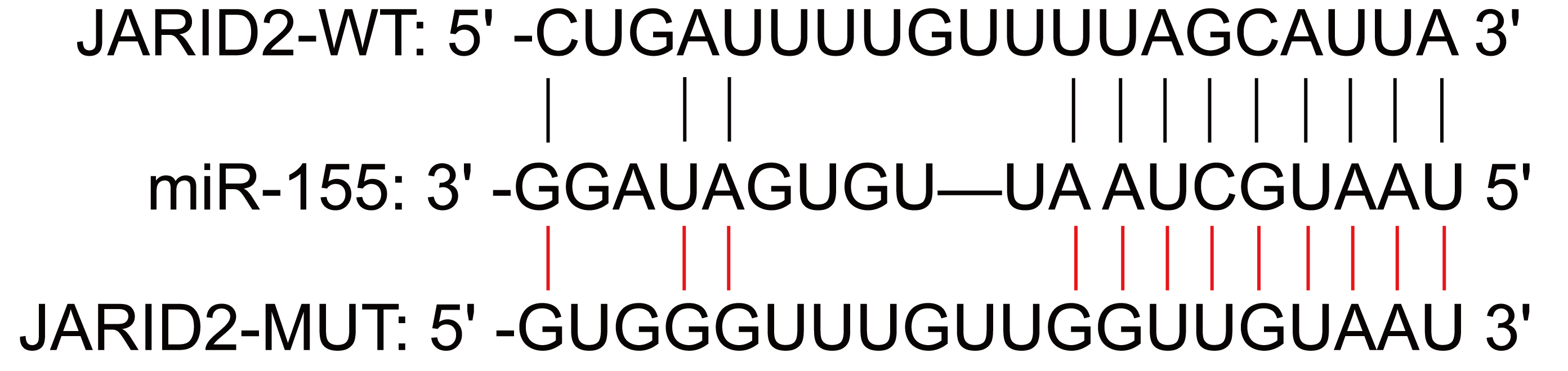

Supplement: S4 Raw data — (ZIP) [file pone.0296671.s005.zip › images/4A.tif]

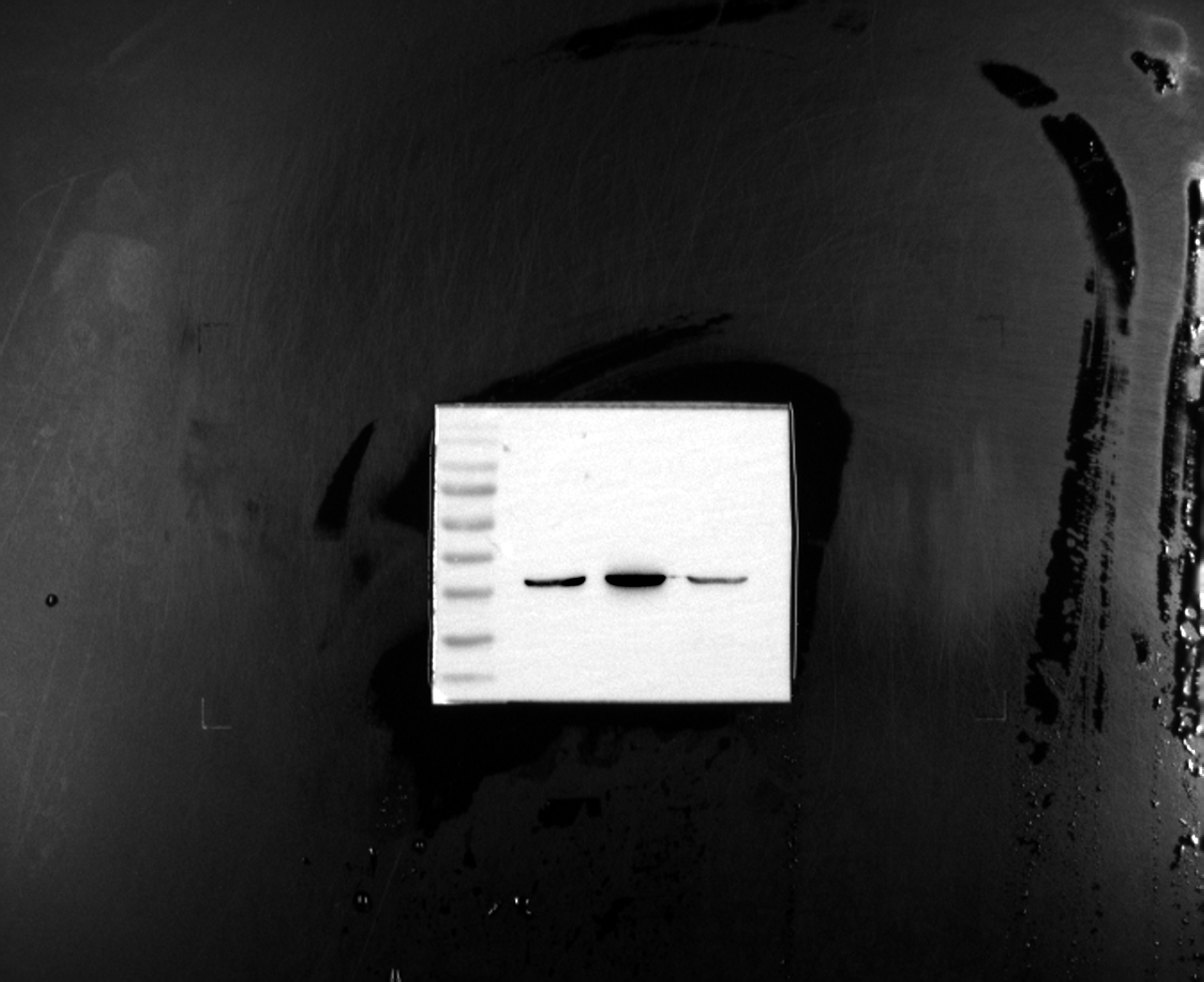

Supplement: S4 Raw data — (ZIP) [file pone.0296671.s005.zip › images/4C/1 JAPID2.tif]

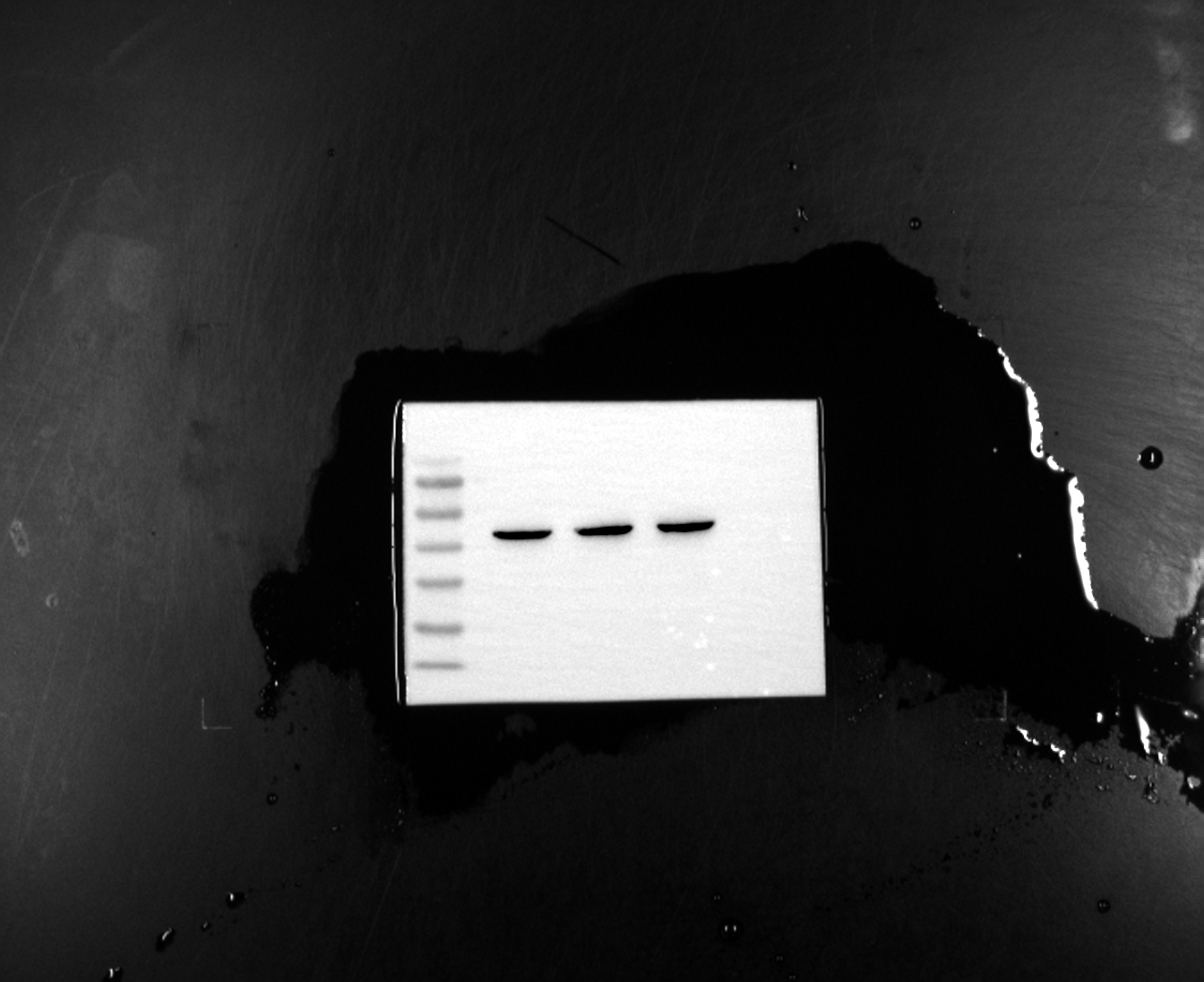

Supplement: S4 Raw data — (ZIP) [file pone.0296671.s005.zip › images/4C/2 β-actin.tif]

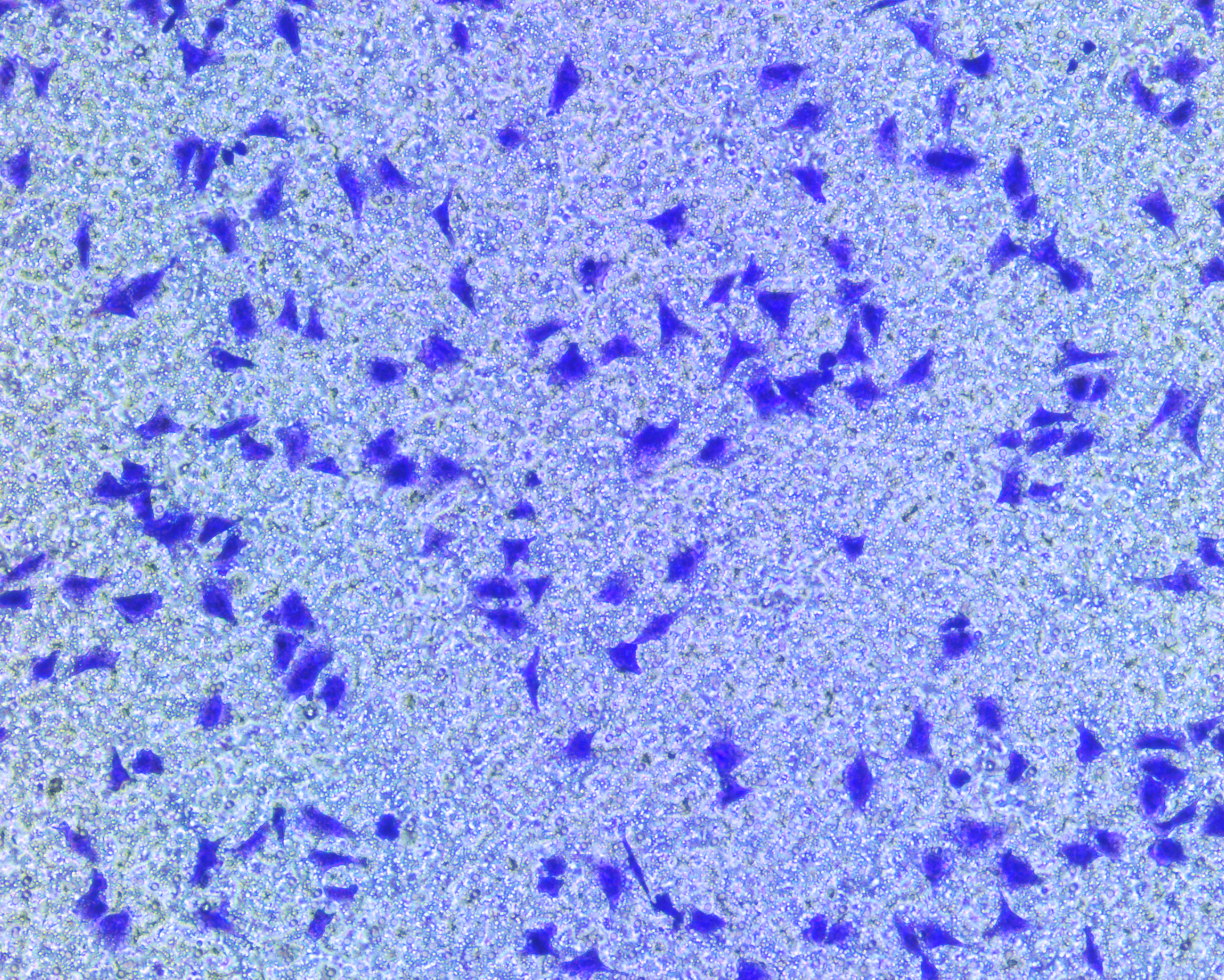

Supplement: S5 Raw data — (ZIP) [file pone.0296671.s006.zip › images/5D/miR-155mimic+PC-JARID2.tif]

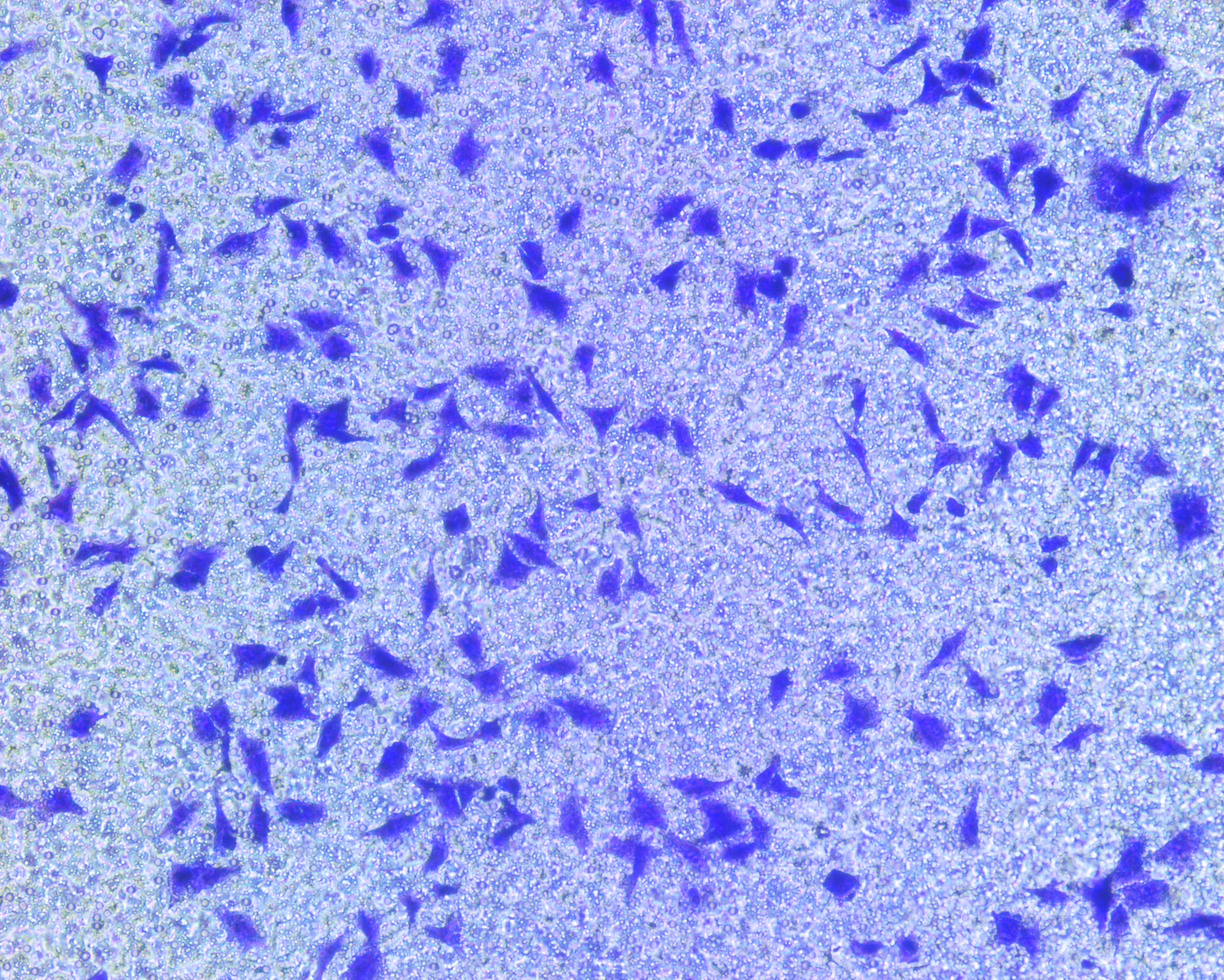

Supplement: S5 Raw data — (ZIP) [file pone.0296671.s006.zip › images/5D/miR-155mimic+PC-NC.tif]

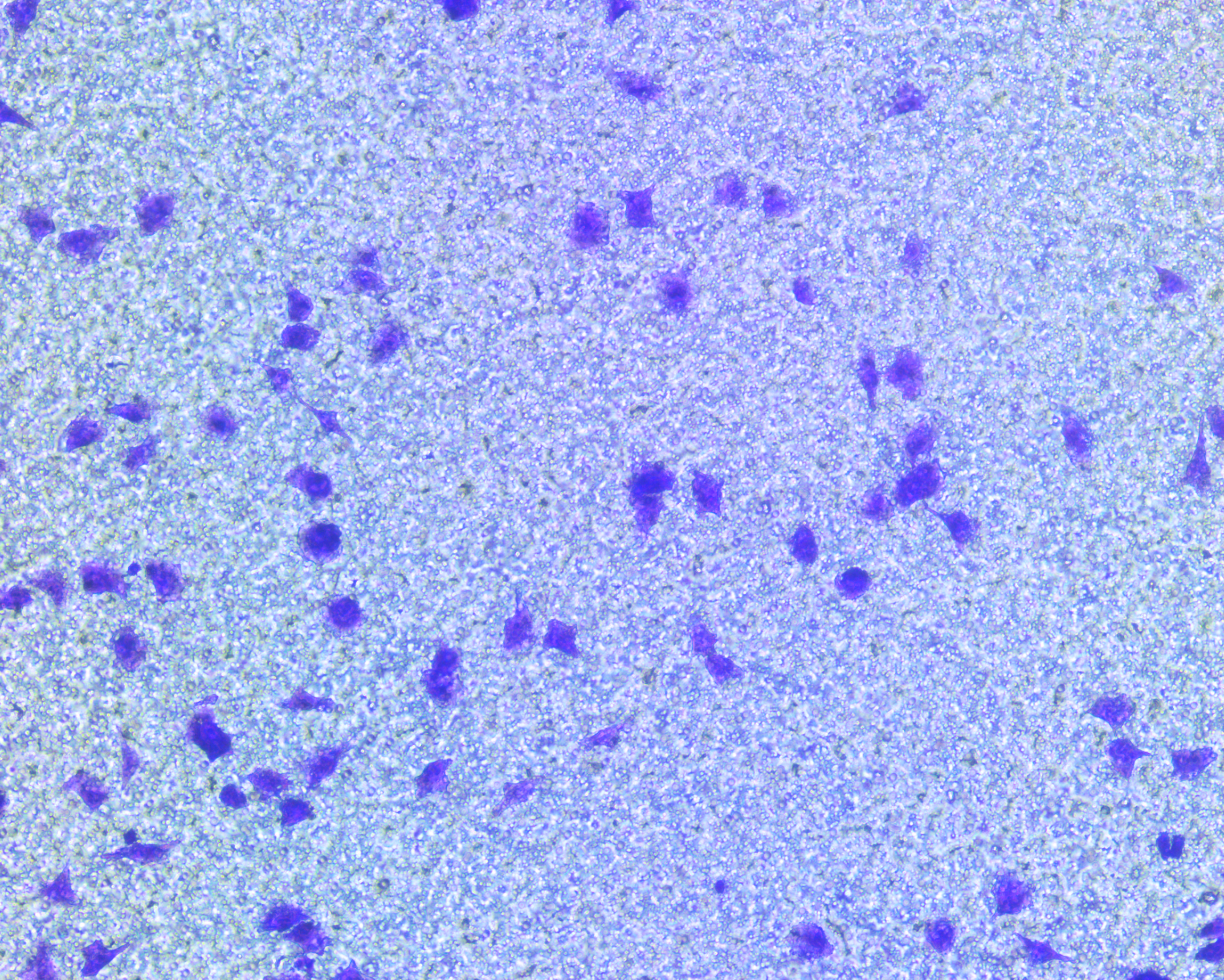

Supplement: S5 Raw data — (ZIP) [file pone.0296671.s006.zip › images/5D/NC.tif]

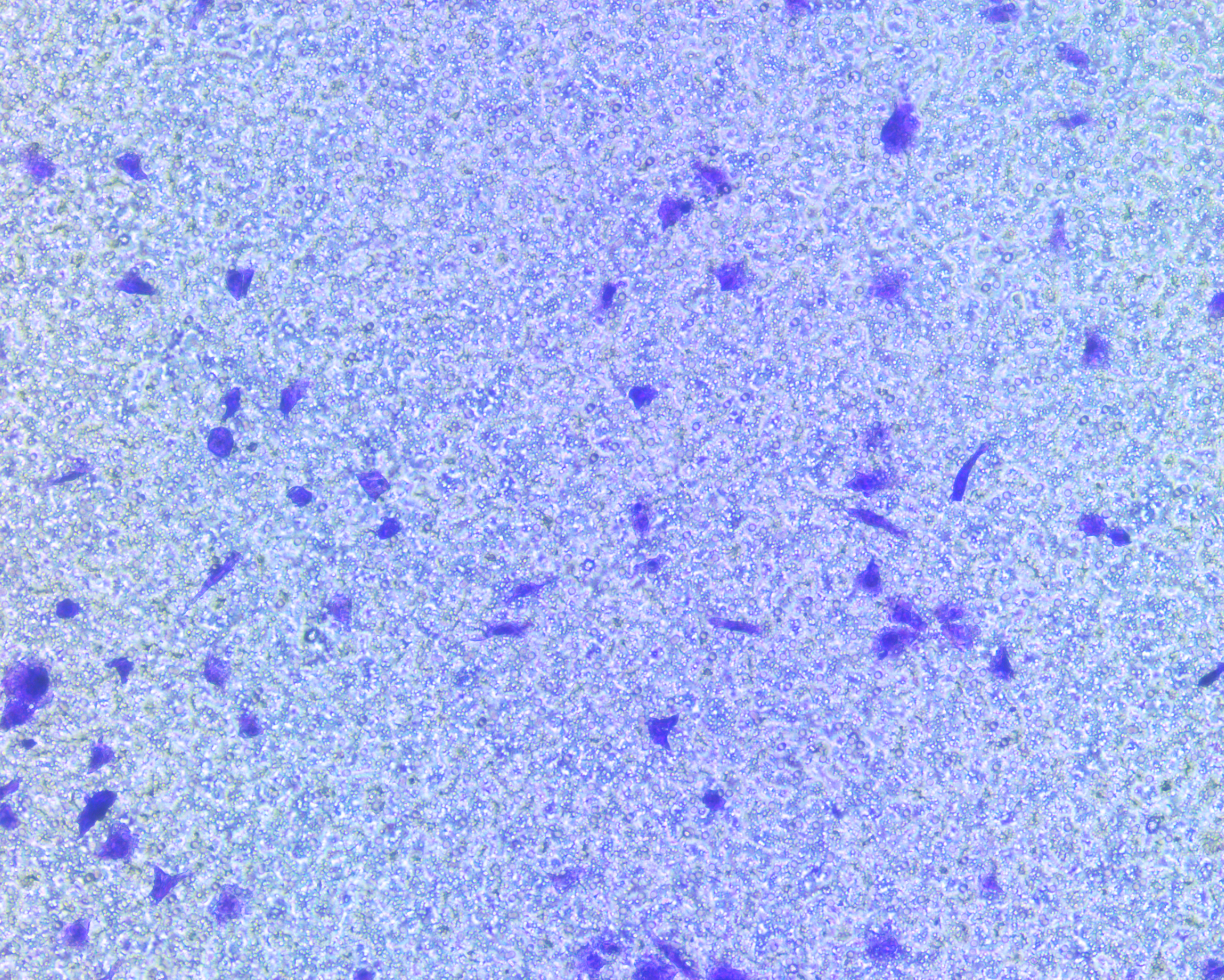

Supplement: S5 Raw data — (ZIP) [file pone.0296671.s006.zip › images/5D/NCmimic+PC-JARID2.tif]

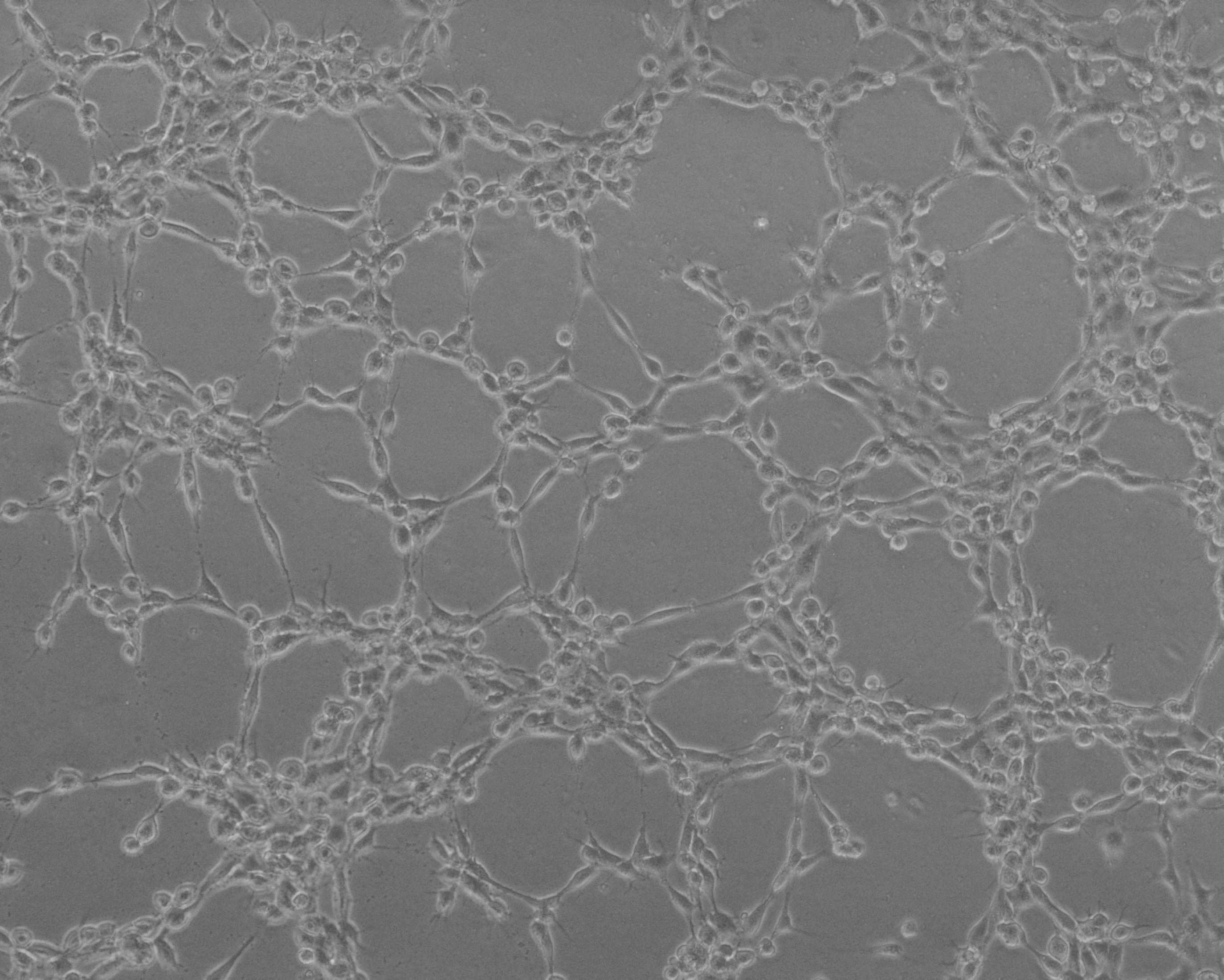

Supplement: S5 Raw data — (ZIP) [file pone.0296671.s006.zip › images/5E/miR-155mimic+PC-JARID2.tif]

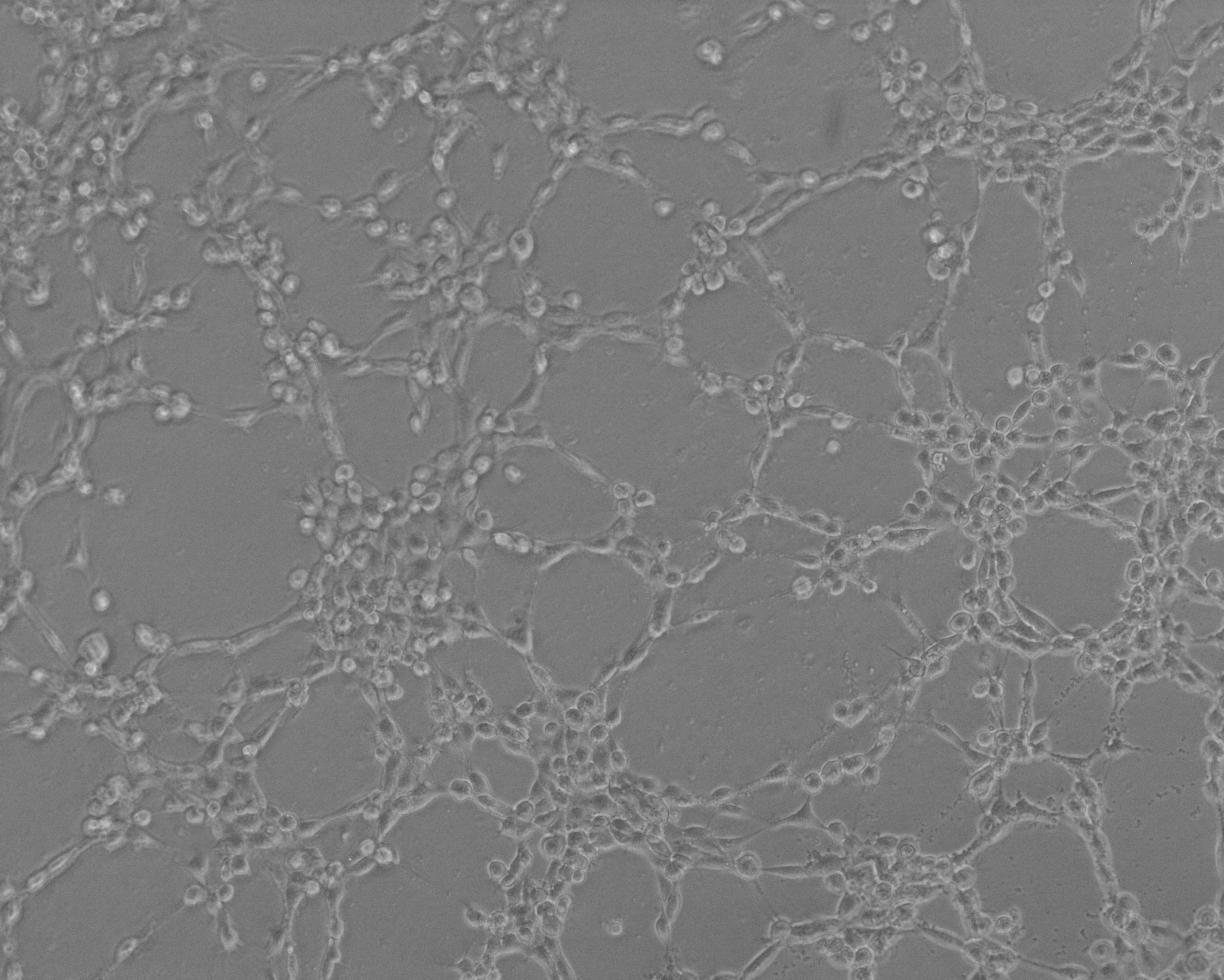

Supplement: S5 Raw data — (ZIP) [file pone.0296671.s006.zip › images/5E/miR-155mimic+PC-NC.tif]

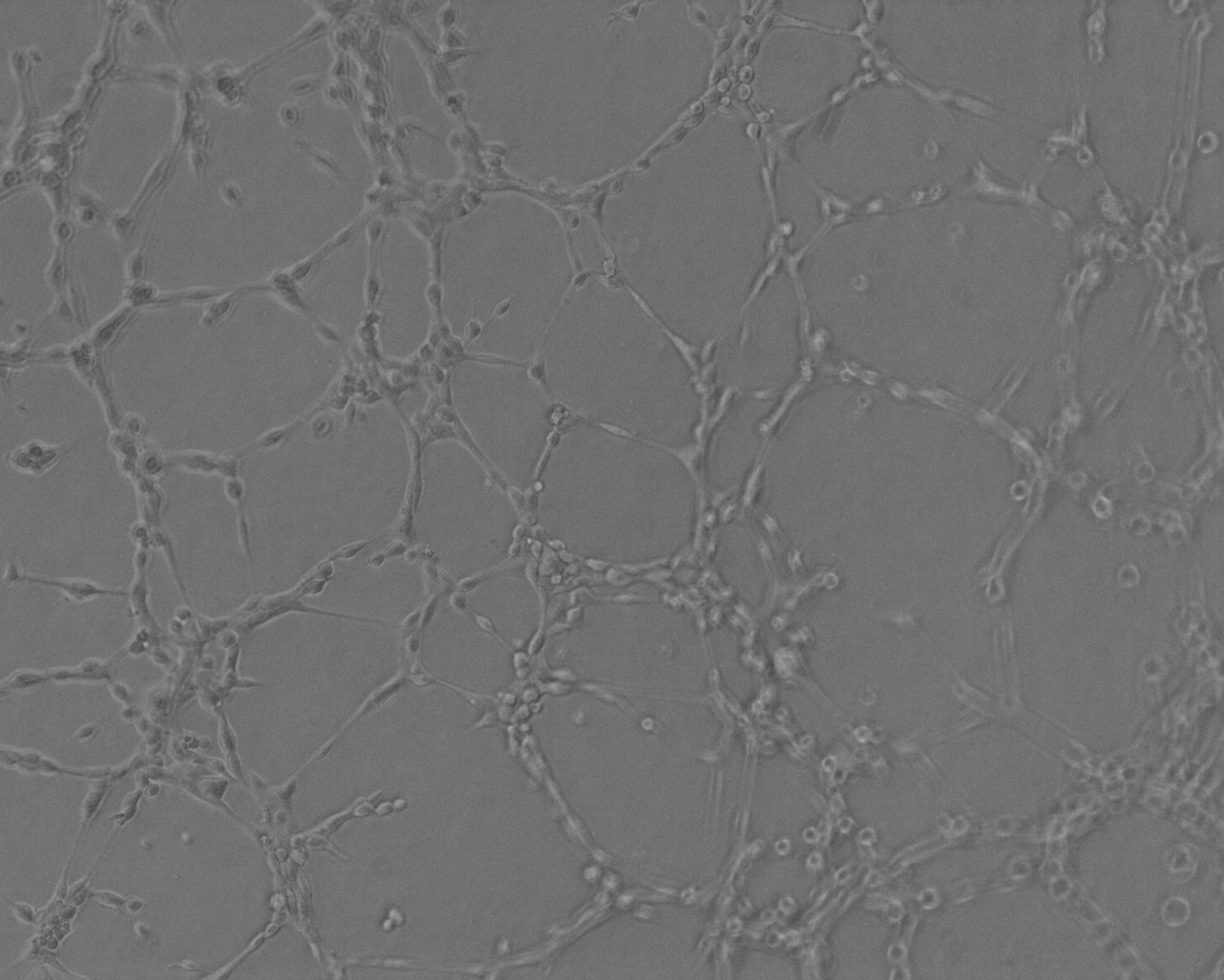

Supplement: S5 Raw data — (ZIP) [file pone.0296671.s006.zip › images/5E/NC.tif]

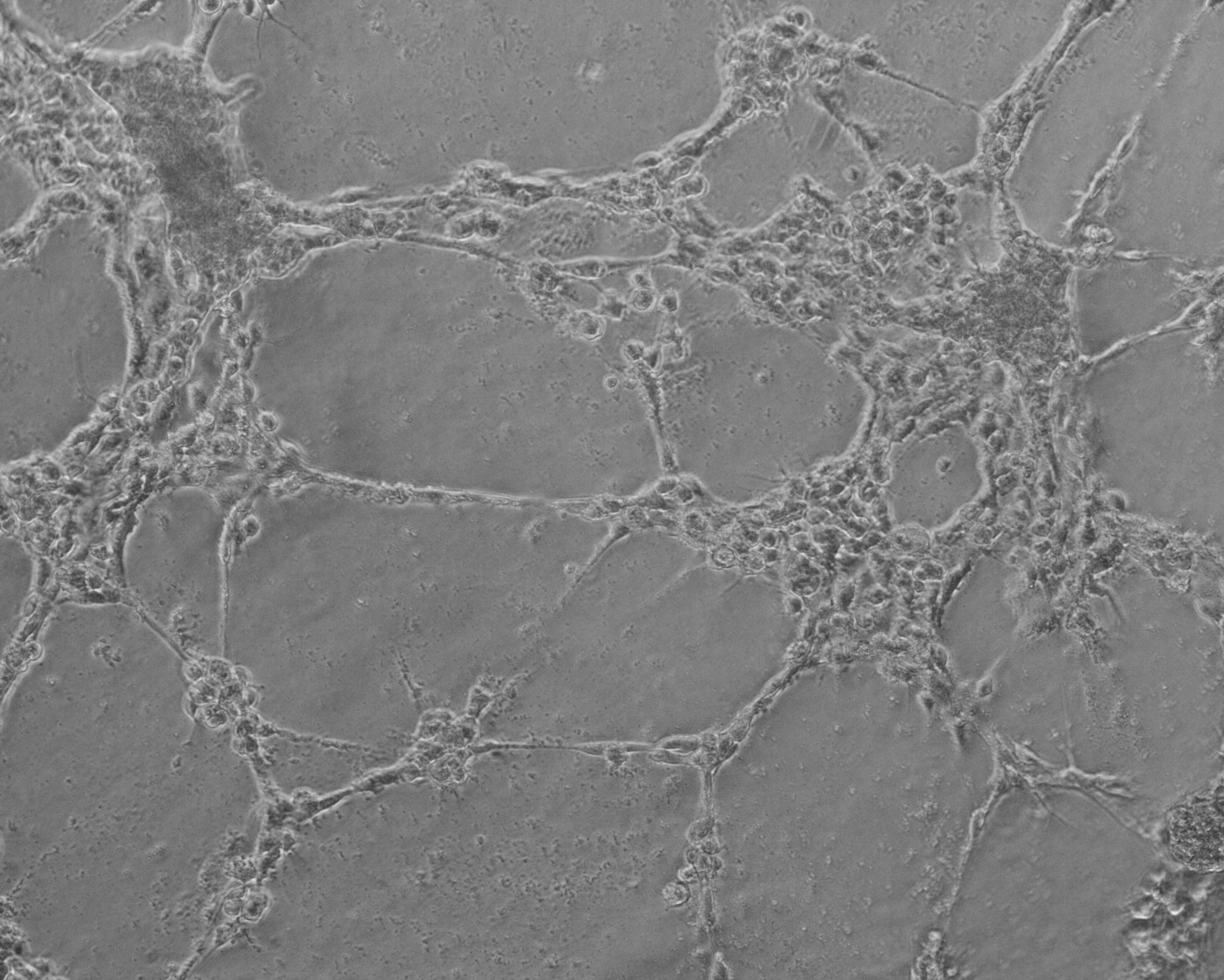

Supplement: S5 Raw data — (ZIP) [file pone.0296671.s006.zip › images/5E/NCmimic+PC-JARID2.tif]

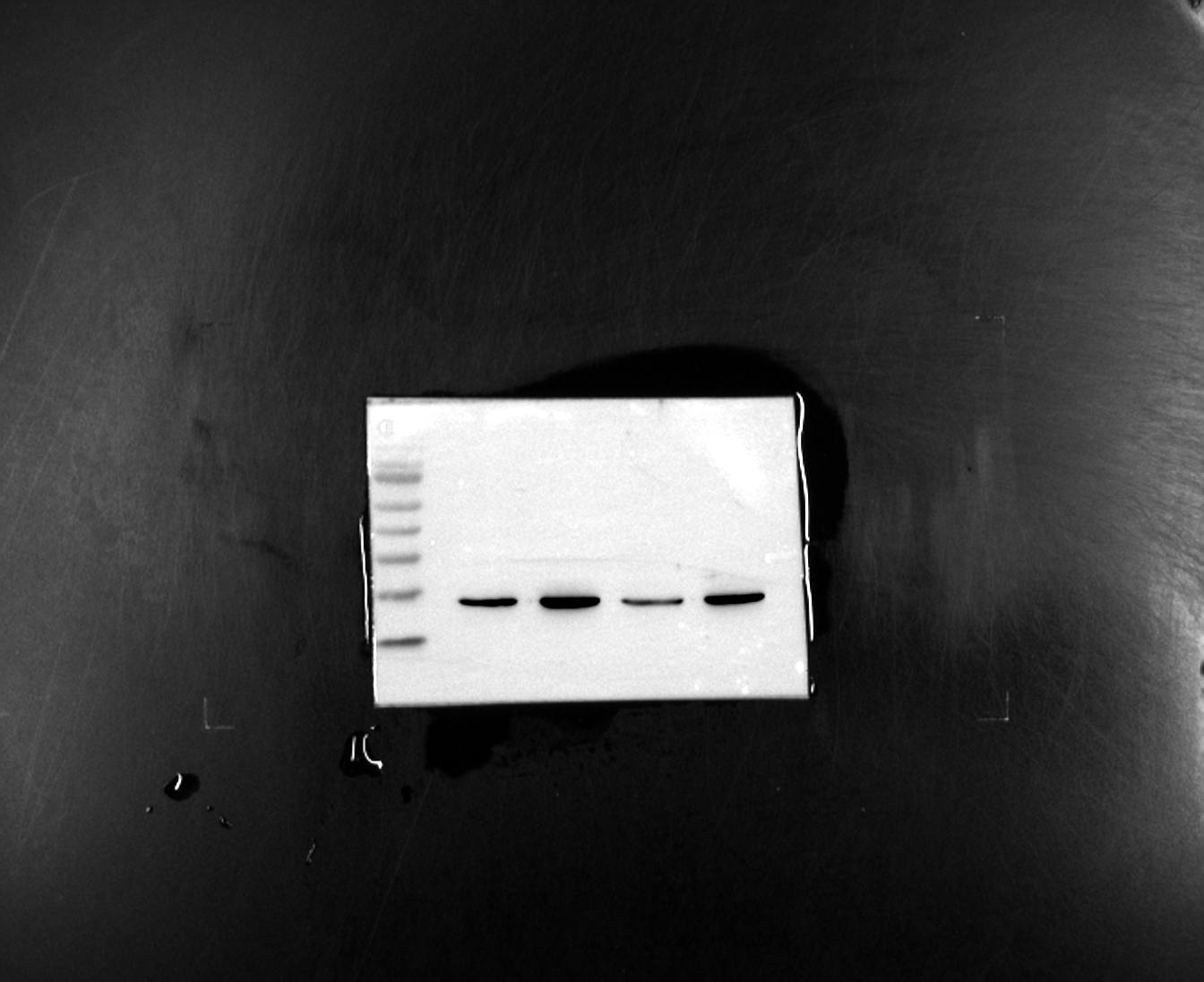

Supplement: S5 Raw data — (ZIP) [file pone.0296671.s006.zip › images/5G/1 VEGF.tif]

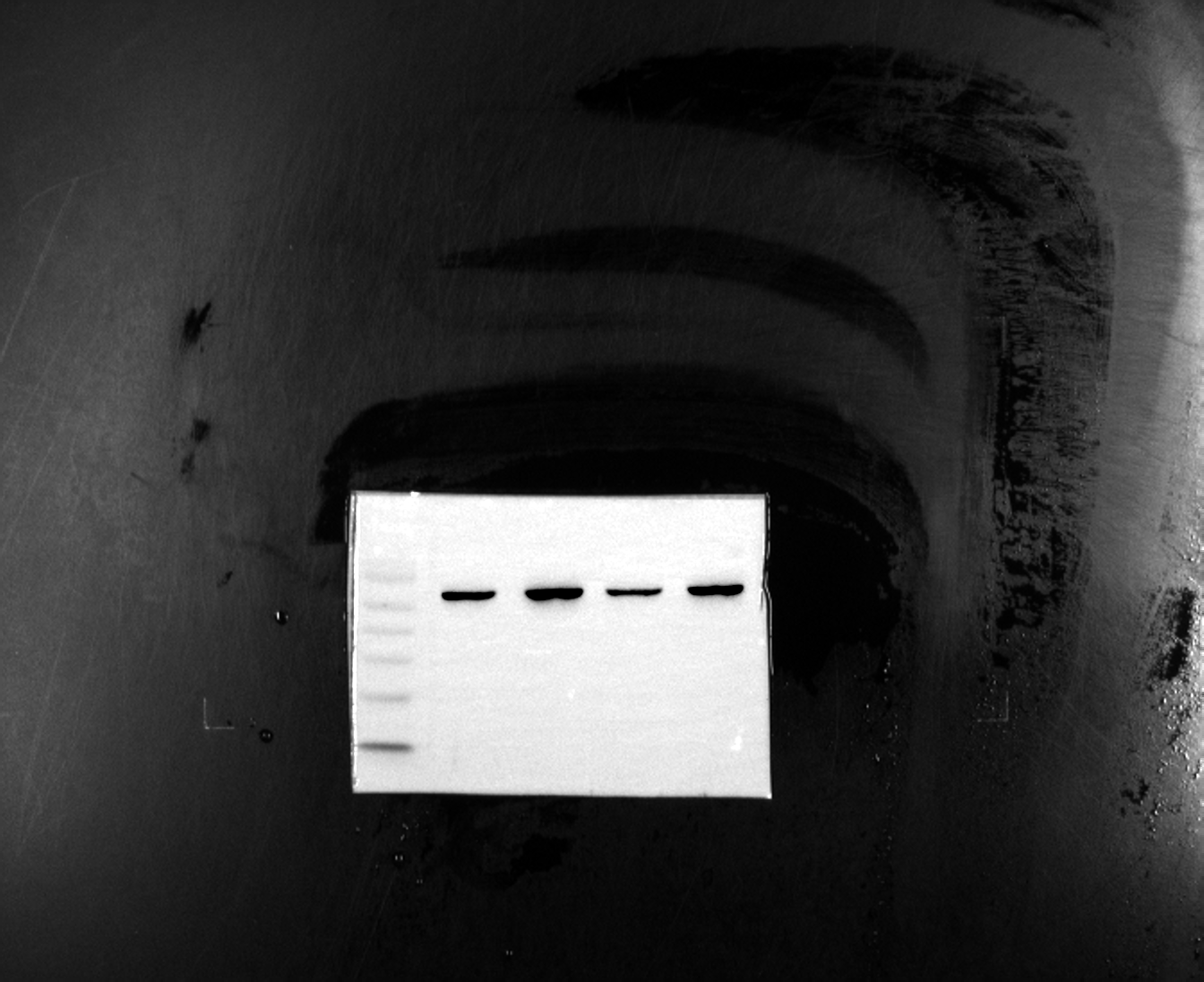

Supplement: S5 Raw data — (ZIP) [file pone.0296671.s006.zip › images/5G/2 MMP-2.tif]

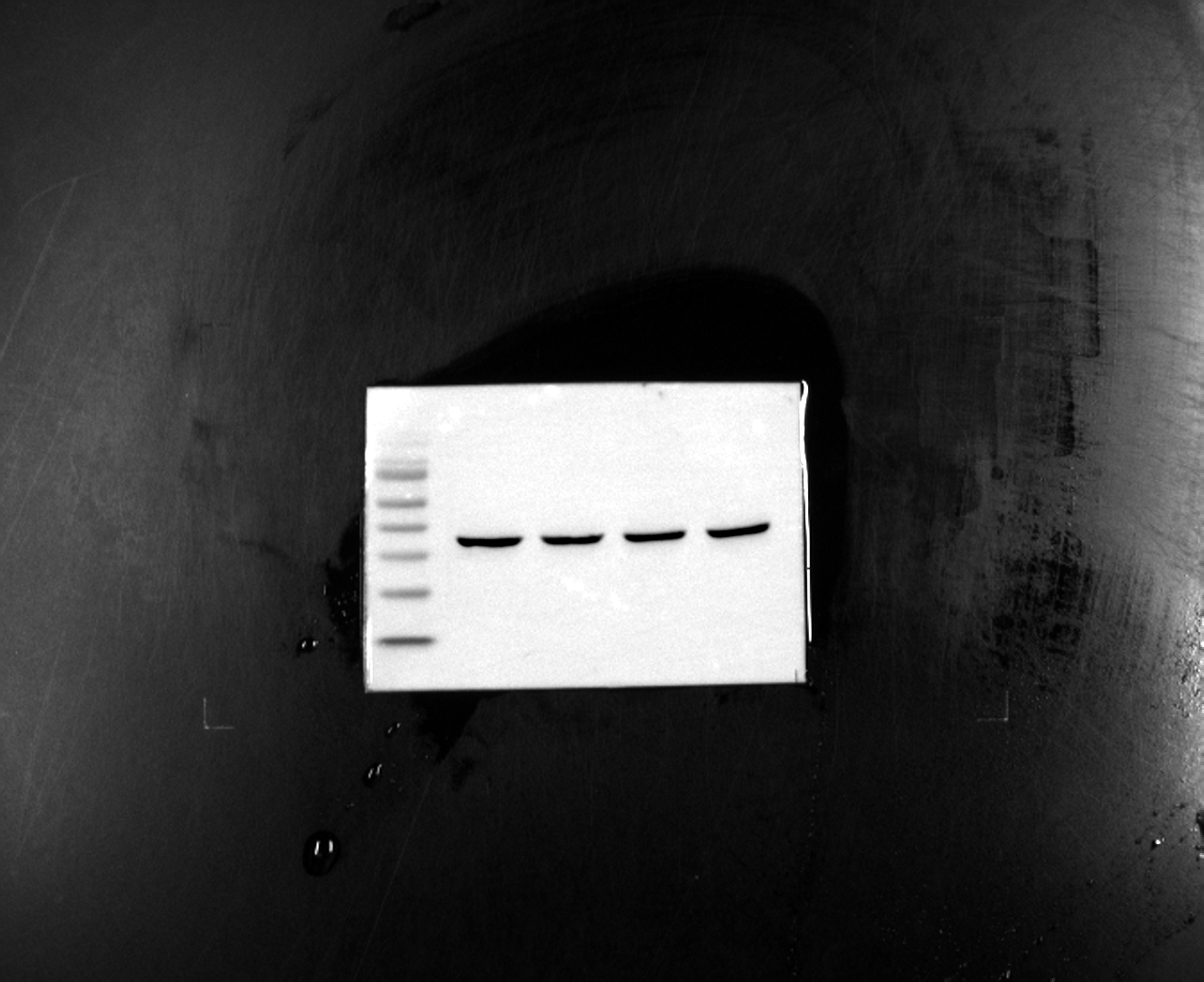

Supplement: S5 Raw data — (ZIP) [file pone.0296671.s006.zip › images/5G/3 GAPDH.tif]

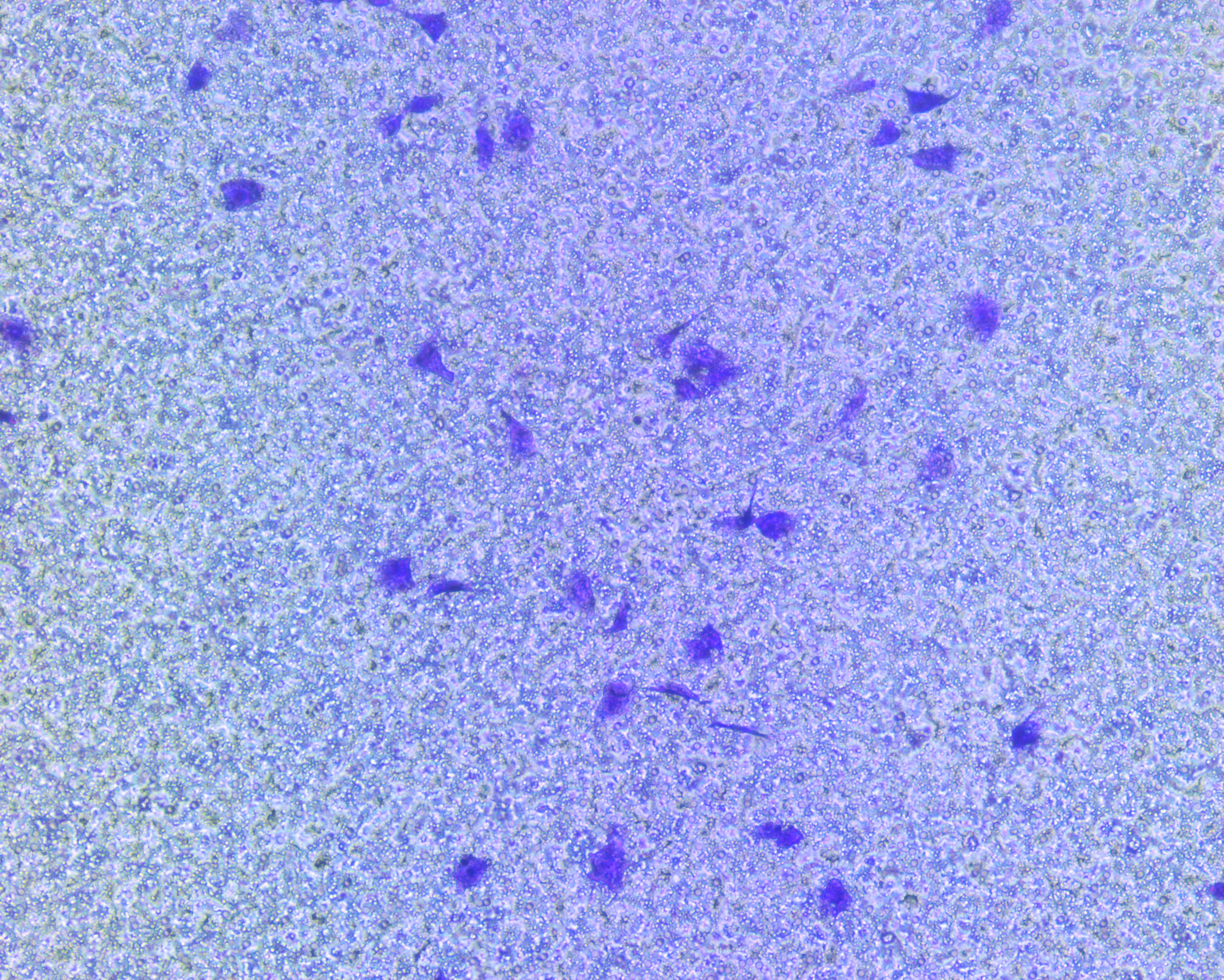

Supplement: S6 Raw data — (ZIP) [file pone.0296671.s007.zip › images/6D/miR-155 inhibitor+si-NC.tif]

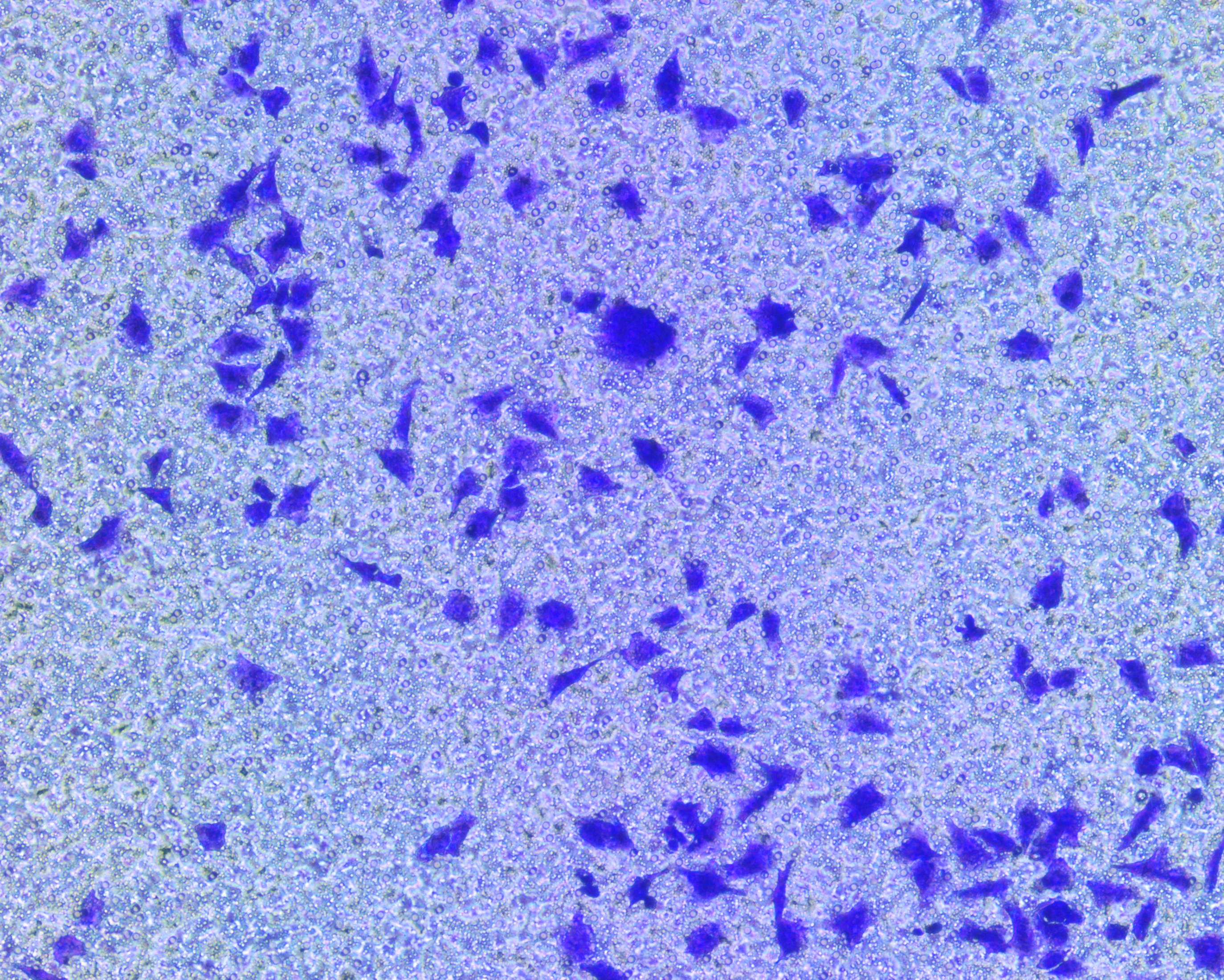

Supplement: S6 Raw data — (ZIP) [file pone.0296671.s007.zip › images/6D/miR-155inhibitor+si-JARID2.tif]

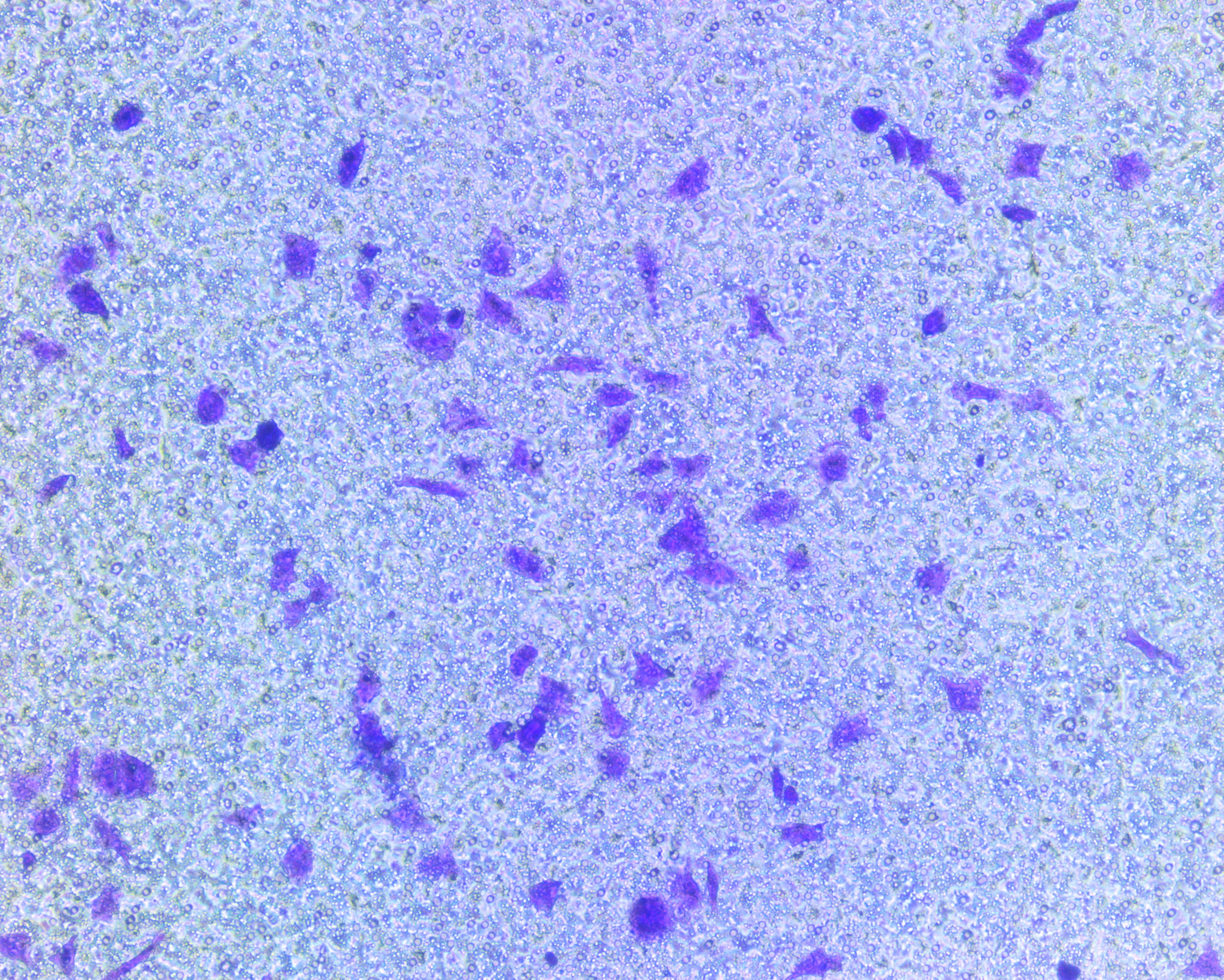

Supplement: S6 Raw data — (ZIP) [file pone.0296671.s007.zip › images/6D/NC.tif]

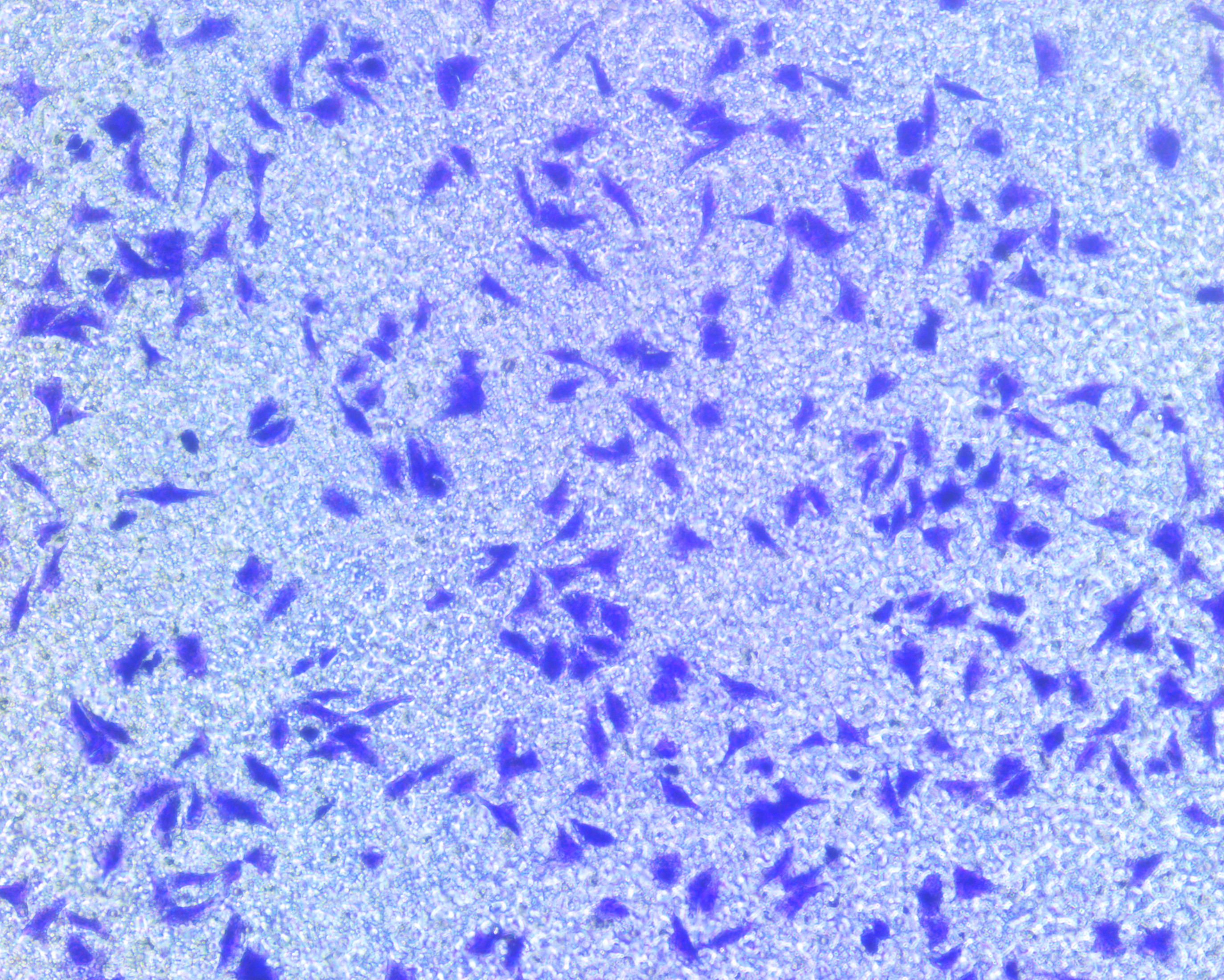

Supplement: S6 Raw data — (ZIP) [file pone.0296671.s007.zip › images/6D/NCinhibitor+si-JARID2.tif]

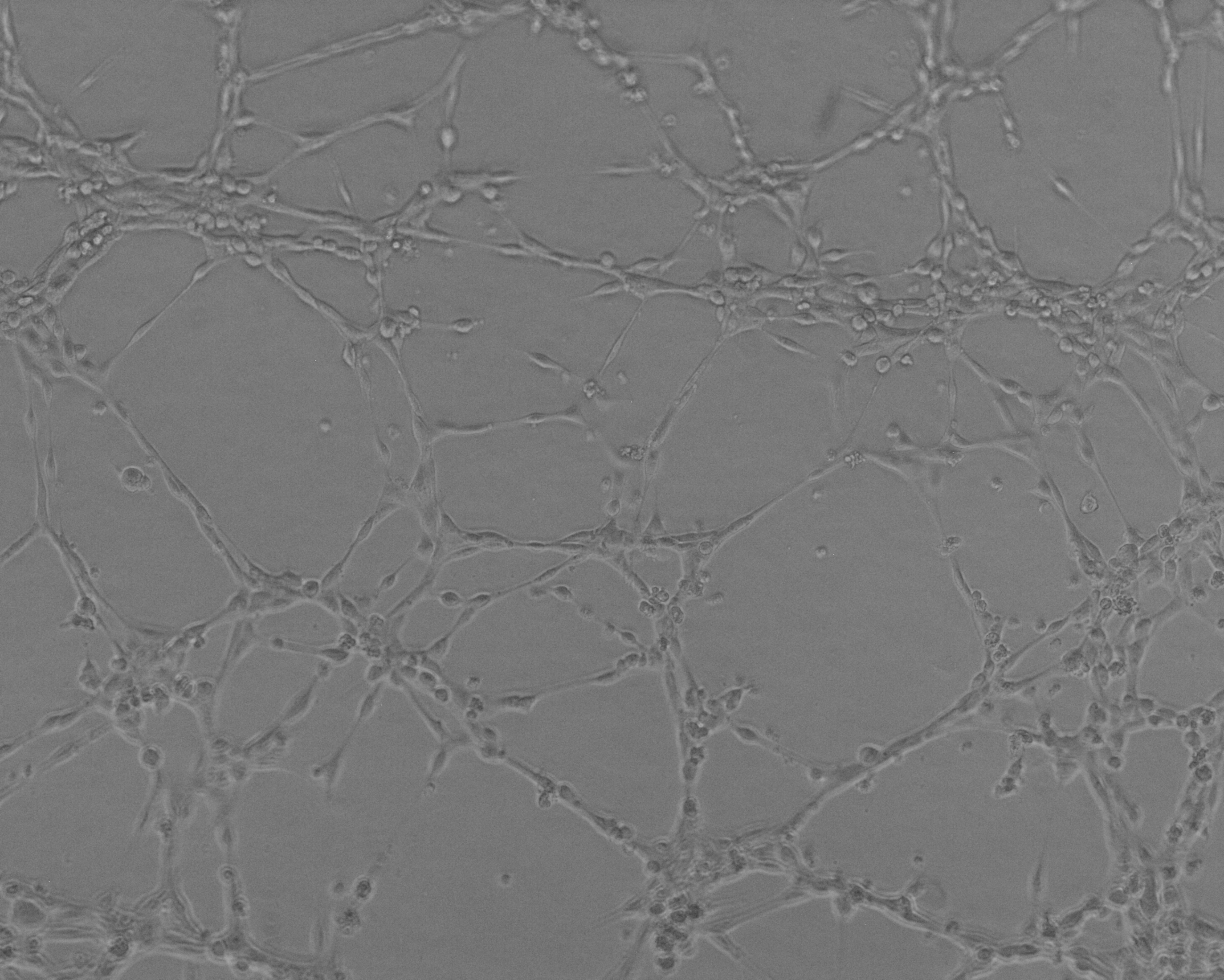

Supplement: S6 Raw data — (ZIP) [file pone.0296671.s007.zip › images/6E/miR-155inhibitor+si-JARID2.tif]

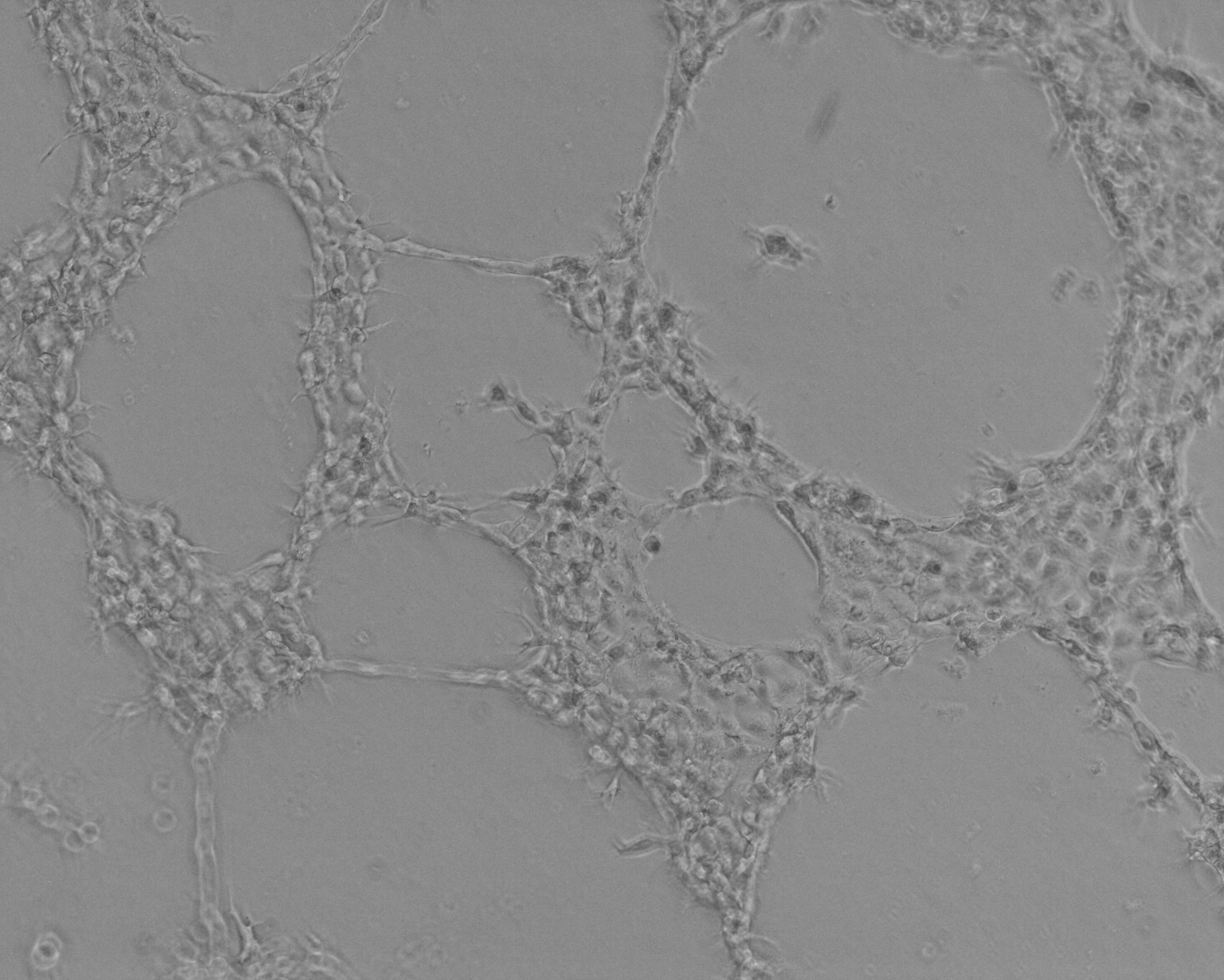

Supplement: S6 Raw data — (ZIP) [file pone.0296671.s007.zip › images/6E/miR-155inhibitor+si-NC.tif]

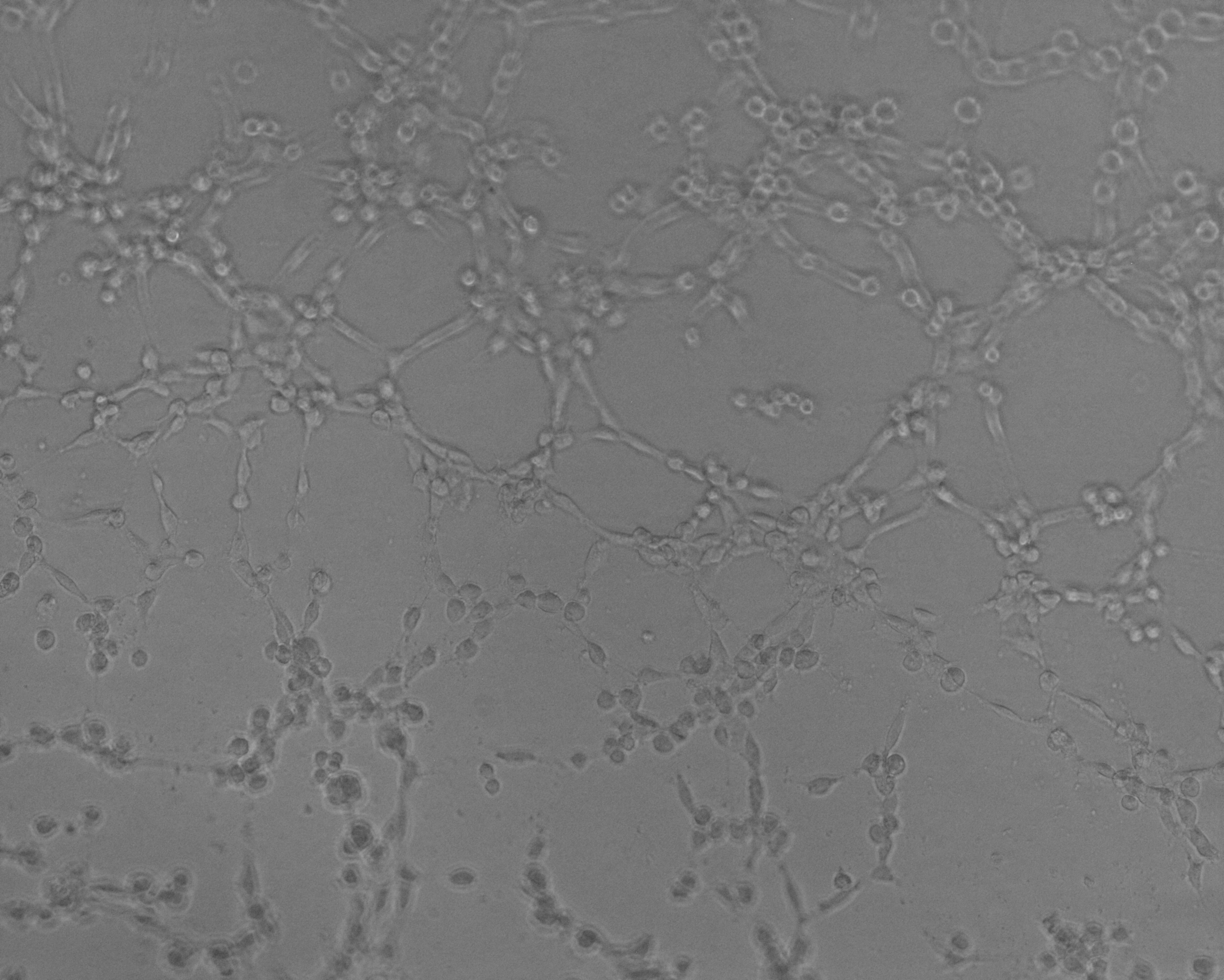

Supplement: S6 Raw data — (ZIP) [file pone.0296671.s007.zip › images/6E/NC inhibitor+si-JARID2.tif]

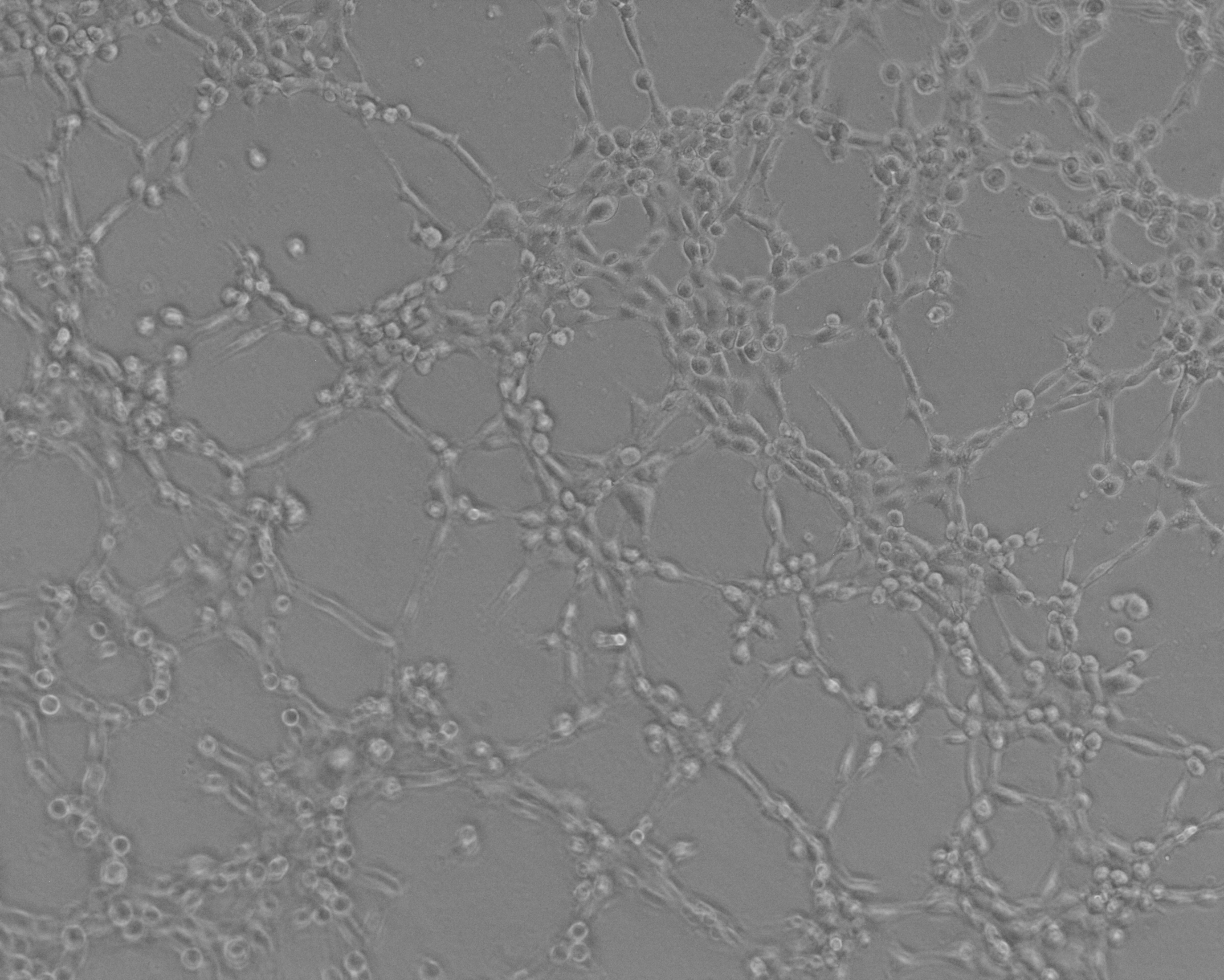

Supplement: S6 Raw data — (ZIP) [file pone.0296671.s007.zip › images/6E/NC.tif]

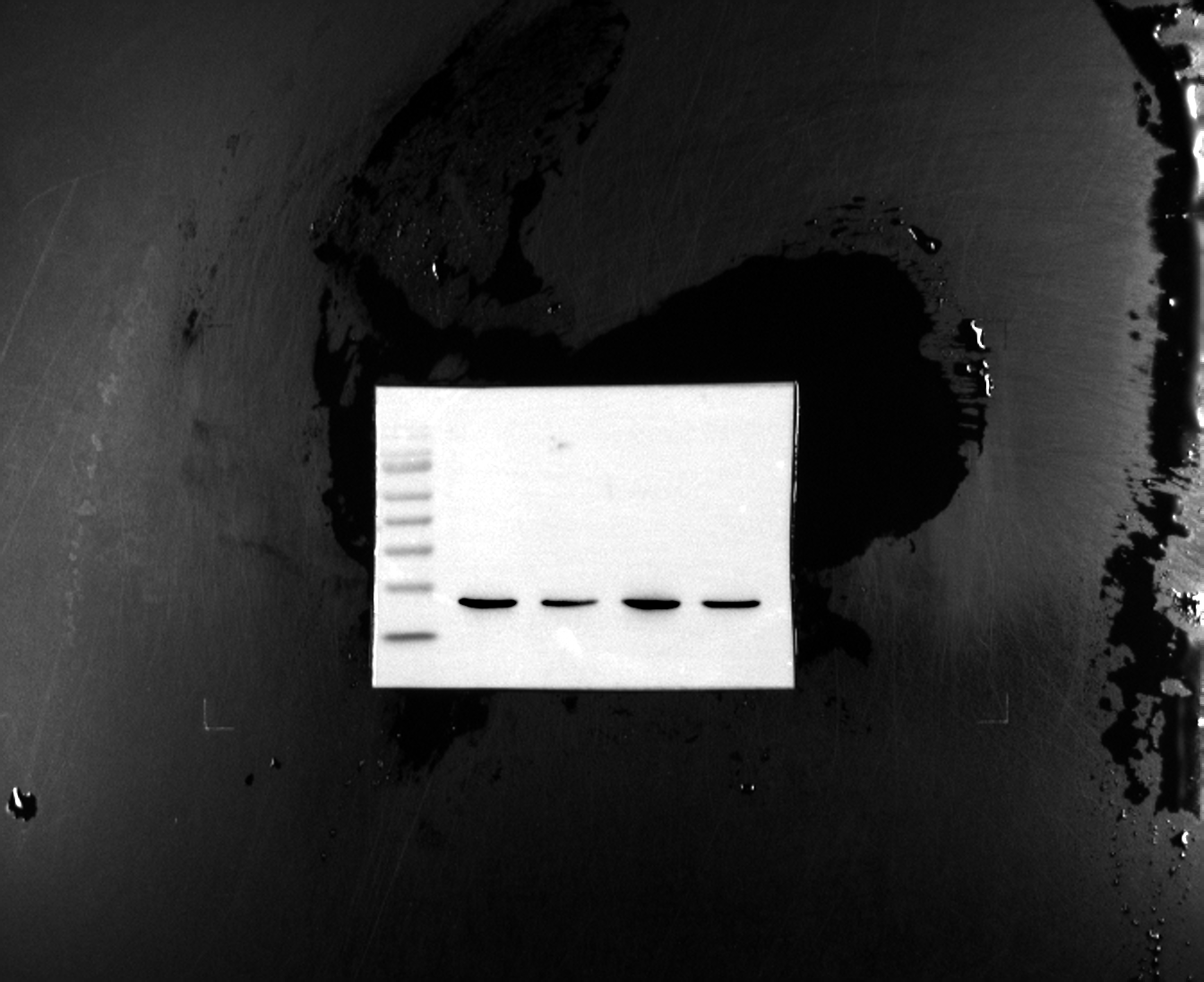

Supplement: S6 Raw data — (ZIP) [file pone.0296671.s007.zip › images/6G/1 VEGF.tif]

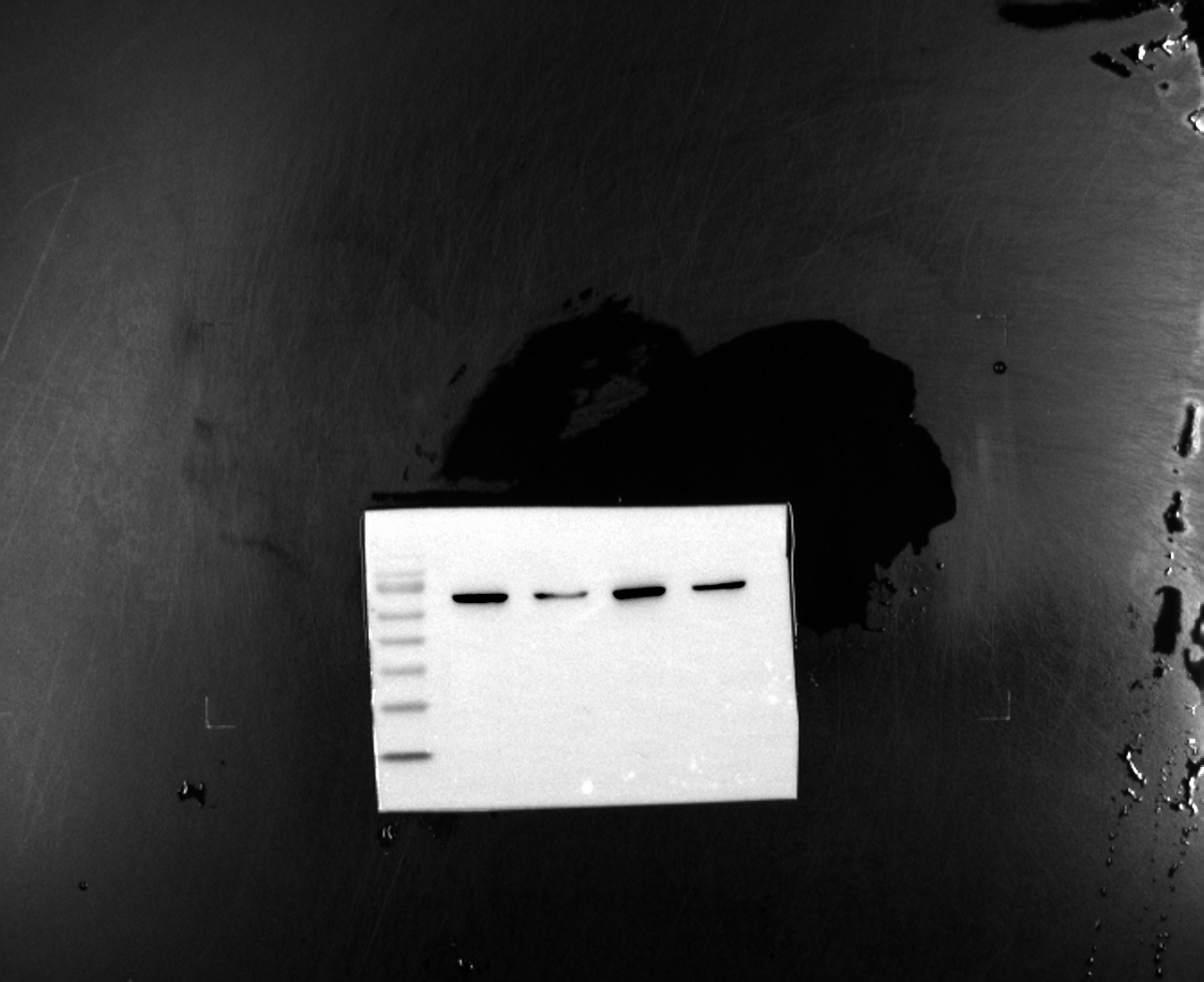

Supplement: S6 Raw data — (ZIP) [file pone.0296671.s007.zip › images/6G/2 MMP-2.tif]

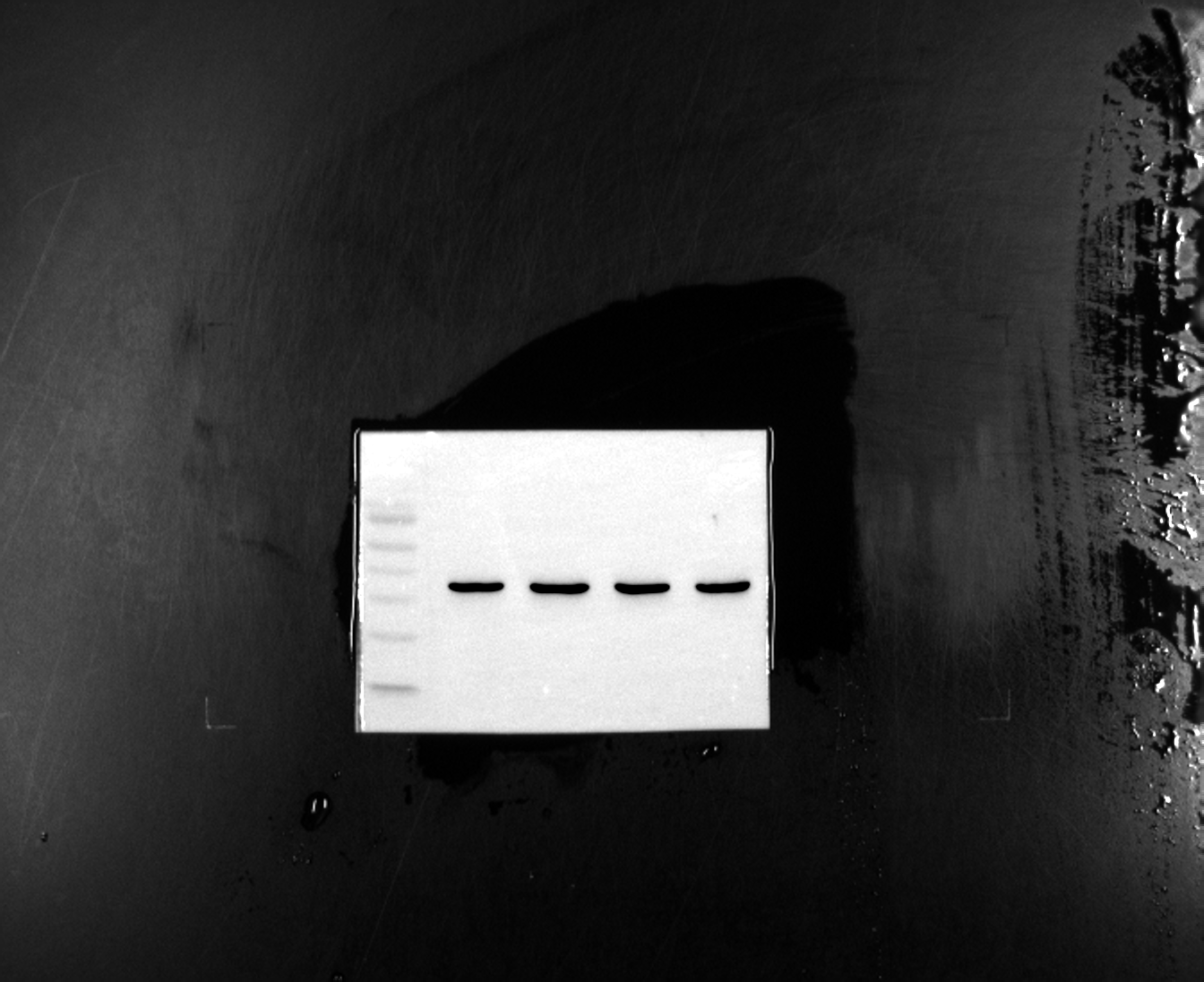

Supplement: S6 Raw data — (ZIP) [file pone.0296671.s007.zip › images/6G/3 GAPDH.tif]

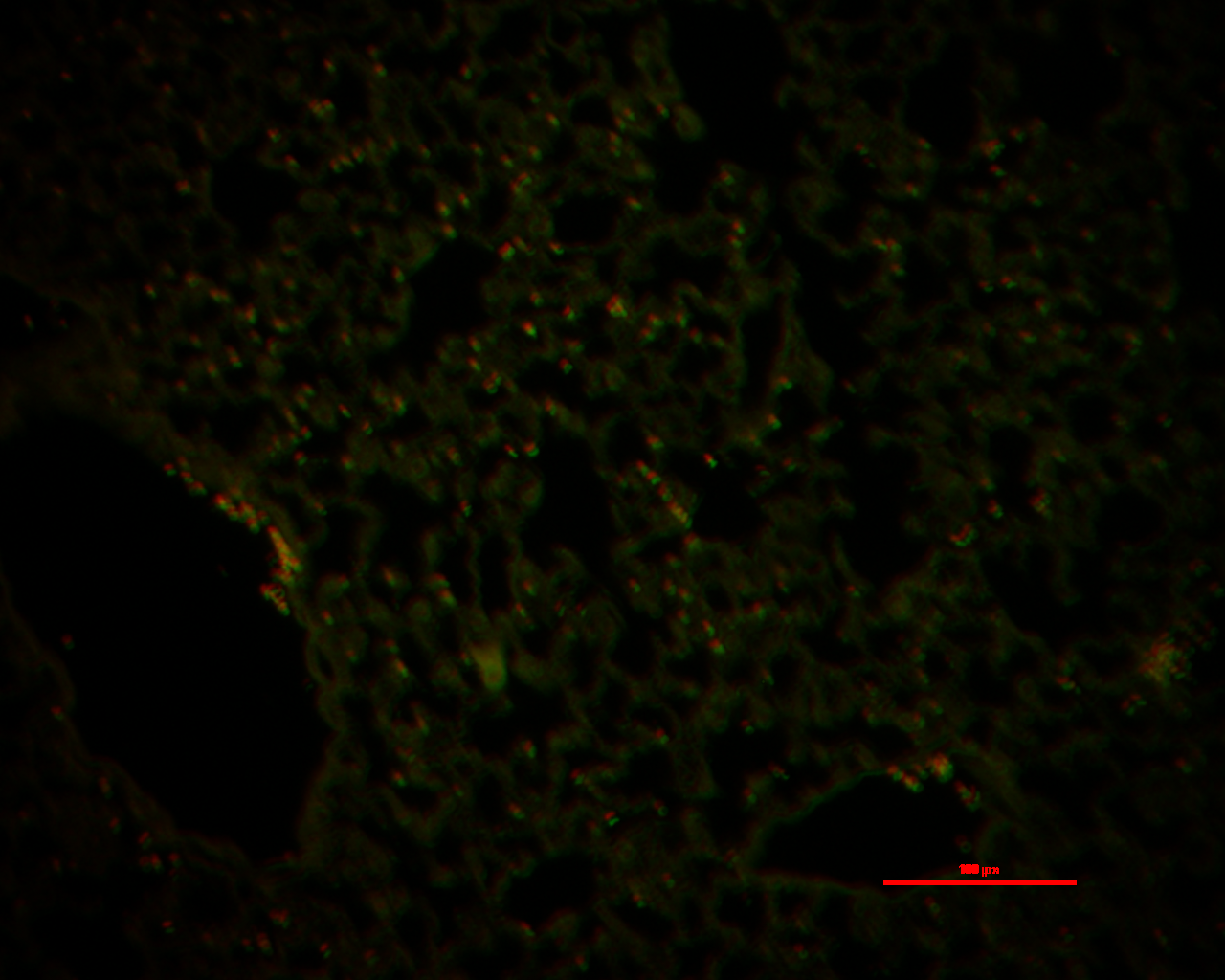

Supplement: S8 Raw data — (ZIP) [file pone.0296671.s009.zip › images/8A/miR-155mimic+pc-JARID2.tif]

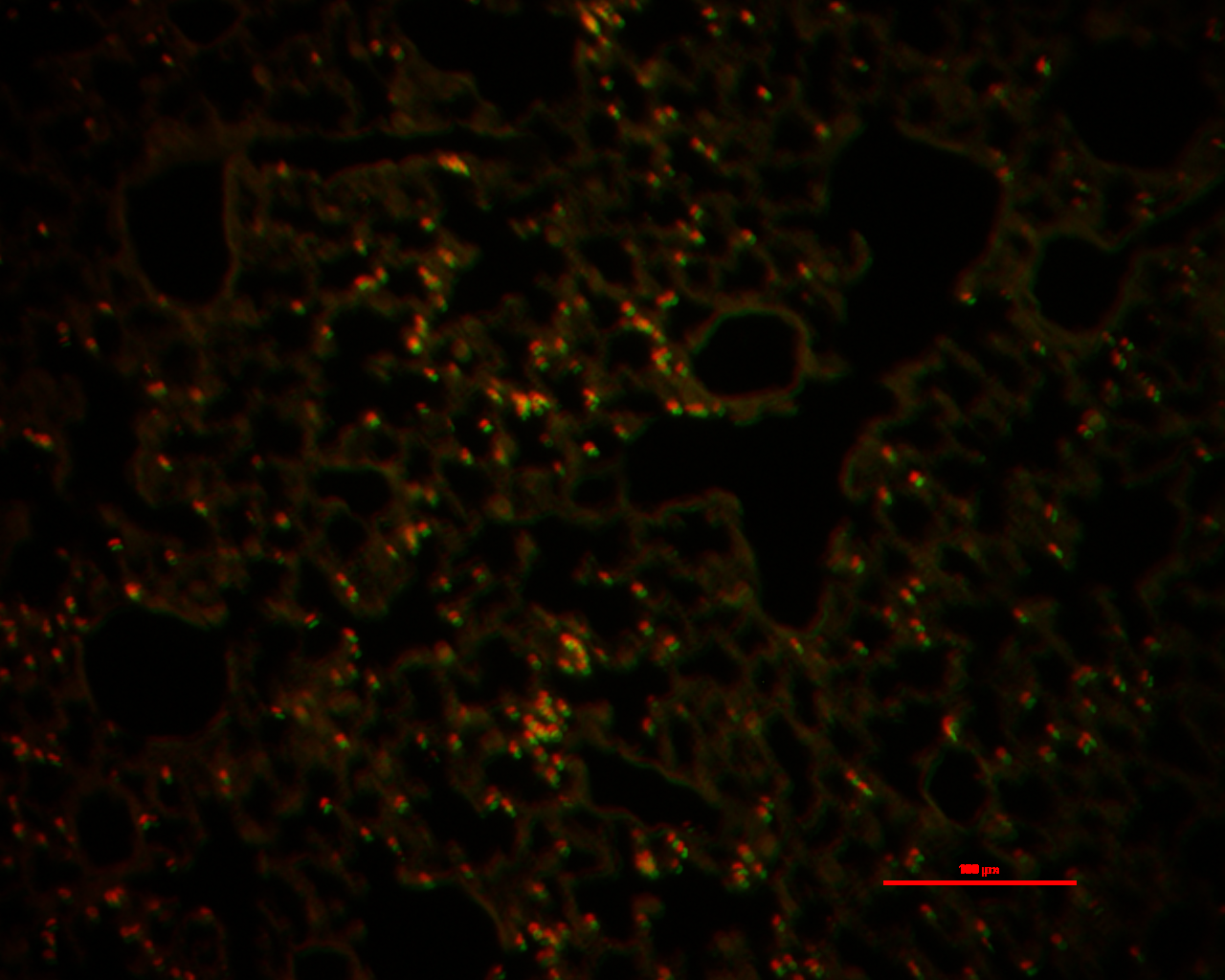

Supplement: S8 Raw data — (ZIP) [file pone.0296671.s009.zip › images/8A/miR-155mimic+pc-NC.tif]

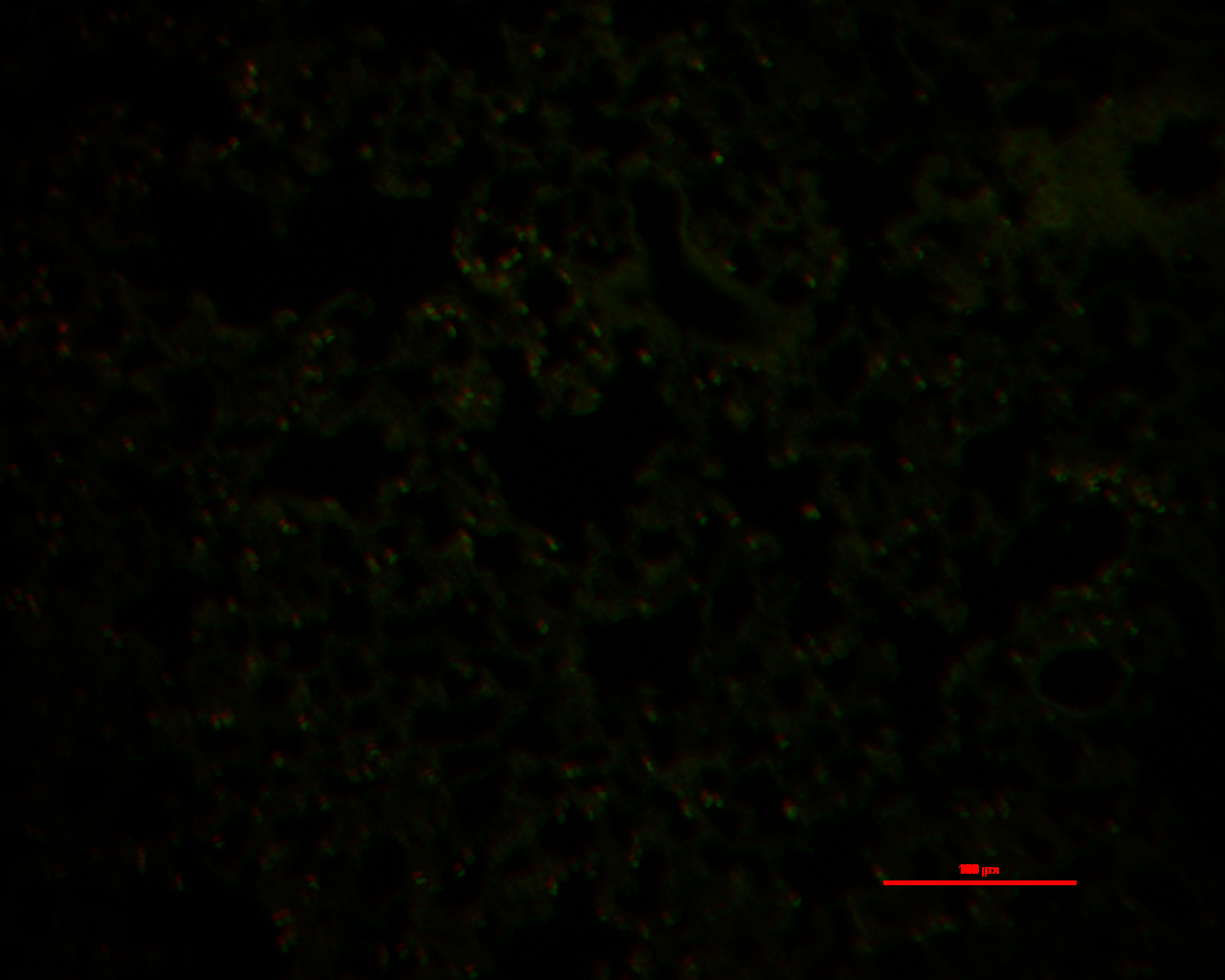

Supplement: S8 Raw data — (ZIP) [file pone.0296671.s009.zip › images/8A/NC mimic+pc-JARID2.tif]

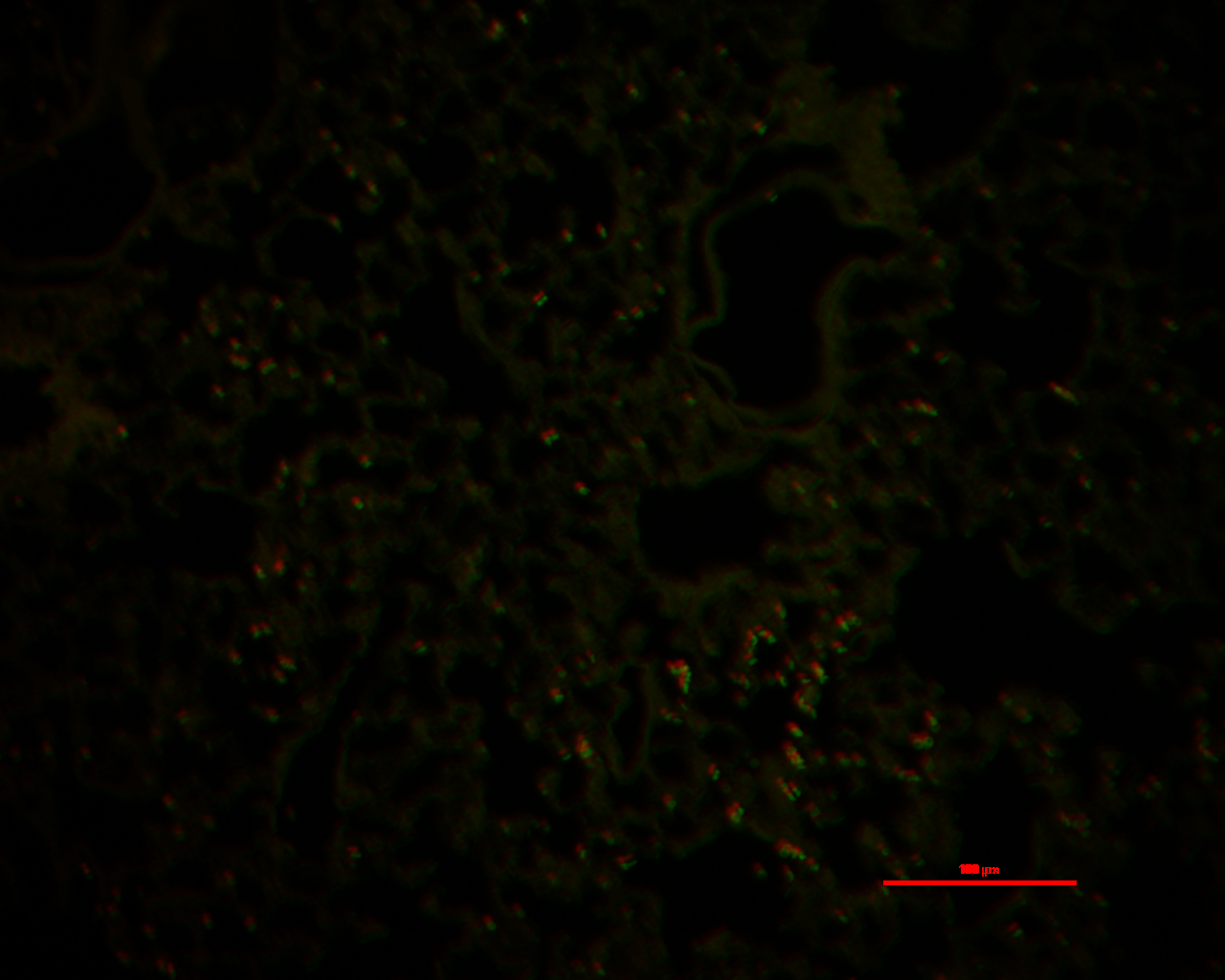

Supplement: S8 Raw data — (ZIP) [file pone.0296671.s009.zip › images/8A/NC.tif]
